# Supplementary material for: Accelerating fragment-based drug discovery using grand canonical nonequilibrium candidate Monte Carlo
Source: Nat Commun. 2025 Jul 4;16:6198. doi: 10.1038/s41467-025-60561-3 (PMC12227770; doi:10.1038/s41467-025-60561-3)
Supplement: Supplementary file 1 — Supplementary Information [file 41467_2025_60561_MOESM1_ESM.pdf]

Supporting Information: Accelerating Fragment Based Drug Discovery using Grand Canonical Nonequilibrium Candidate Monte Carlo

William G. Poole[1], Marley L. Samways[1,3], Davide Branduardi[2], Richard D. Taylor[3], Marcel L. Verdonk[2], Jonathan W. Essex[1*]

[1*]School of Chemistry, University of Southampton, Southampton, SO17 1BJ, United Kingdom.
[2]Astex Pharmaceuticals, 436 Cambridge Science Park, Milton Road, Cambridge, CB4 0QA, United Kingdom.
[3]UCB, 216 Bath Road, Slough, SL1 3WE, United Kingdom.

*Corresponding author(s). E-mail(s): j.w.essex@soton.ac.uk;
Contributing authors: wp1g16@soton.ac.uk;

1 Supplementary Discussion: GCNCMC Theory

1.1 Acceptance Criteria Derivation

Grand canonical Monte Carlo (GCMC) provides a way of exchanging particles between a simulated system and a linked ideal gas reservoir in a theoretically rigorous manner that maintains the overall chemical potential in the total system. A GCMC move can come in two forms, an insertion or a deletion, whereby a molecule is added or removed from the canonical system. In this study, we have combined GCMC moves with Nonequilibrium Candidate Monte Carlo (NCMC) in order to improve acceptance rates. The acceptance criteria for GCNCMC moves are derived below and closely follow that of Melling *et al.*[1]

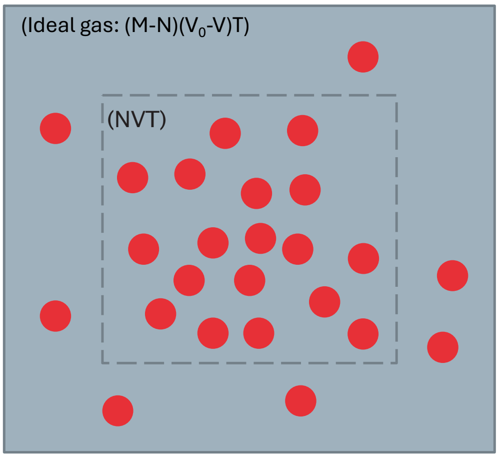

Supplementary Figure 1: Graphical depiction of the grand canonical ensemble. Particles are free to move between the ideal gas and the central NVT system. At equilibrium the chemical potential of the coupled systems is equal.

It is useful to consider the simulated system, *sys*, and the ideal gas reservoir, *gas*, as one large canonical system with a total of M particles in a combined system volume V, where $V = V_{\text{sys}} + V_{\text{gas}}$ (Supplementary Figure 1). For this derivation, we define the number of particles in the simulated system N, such that the total number of particles in the ideal gas is $M - N$. It follows, that the equilibrium probability of a microstate with N particles in the system, and $M - N$ particles in the ideal gas, is given by:

$$\pi_{\text{MVT}}(\mathbf{r}^N, \mathbf{r}^{M-N}, \mathbf{p}^M) = Q^{-1}_{\text{MVT}} h^{-3M} e^{-\beta E(\mathbf{r}^N, \mathbf{r}^{M-N}, \mathbf{p}^M)} d\mathbf{r}^M d\mathbf{p}^M \tag{1}$$

where $\mathbf{r}^N$ and $\mathbf{r}^{M-N}$ are the atomic positions of the particles in the simulated system and ideal gas respectively. $\mathbf{p}^M$ is the momenta of all the particles in the combined system and need not be separated. Q_{MVT} is the canonical partition function of the combined system and E is the total system energy given by:

$$E(\mathbf{r}^N, \mathbf{r}^{M-N}, \mathbf{p}^M) = U(\mathbf{r}^N) + \sum_{i=1}^{M} \frac{|\mathbf{p}_i|^2}{2m} \tag{2}$$

Note that the potential energy term has no dependence on the positions of the ideal gas particles. The momenta is calculated over all particles and therefore does not need separating.

It is now useful to recall the generalized acceptance criteria for a nonequilibrium candidate Monte Carlo (NCMC) move[2]:

$$\frac{A(X|\Lambda_{\text{p}})}{A(\tilde{X}|\tilde{\Lambda}_{\text{p}})} = \frac{P(\tilde{\Lambda}_{\text{p}}|\tilde{x}_T)}{P(\Lambda_{\text{p}}|x_0)} \frac{\alpha(\tilde{X}|\tilde{\Lambda}_{\text{p}})}{\alpha(X|\Lambda_{\text{p}})} \frac{\pi(\tilde{x}_T)}{\pi(x_0)} e^{-\Delta S(X|\Lambda_{\text{p}})} \tag{3}$$

where $P(\Lambda_\mathrm{p}|x_0)$ is the probability of selecting protocol Λ_p and applying it to state x_0. $\alpha(X|\Lambda_\mathrm{p})$ is the cumulative probability of each perturbation step in the forward move and $\Delta S(X|\Lambda_\mathrm{p})$ is called the conditional path action difference. Notations with the $\sim$ represent the reverse move. We will deal with the first three ratios in this equation individually.

Firstly, for an insertion move, a random molecule from the ideal gas is chosen and translated to a random point (with infinitesimal volume, d$\mathbf{r}$) within the simulated system. The probability of selecting this insertion move is given by:

$$P(\Lambda_\mathrm{p}|x_0) = \frac{1}{2}\frac{1}{M-N}\frac{d\mathbf{r}}{V_\mathrm{sys}} \tag{4}$$

where the factor of a half arises because there is a 50% chance of selecting an insertion rather than a deletion. The second term is the probability of selecting one particle at random from $M - N$ ideal gas particles. Lastly, the final term shows that the probability of picking a position in the system is inversely proportional to the volume of the system. The probability of selecting the same move in reverse and returning the particle to the ideal gas is given by:

$$P(\tilde{\Lambda}_\mathrm{p}|\tilde{x}_T) = \frac{1}{2}\frac{1}{N+1}\frac{d\mathbf{r}}{V_\mathrm{gas}} \tag{5}$$

where $N + 1$ is the number of particles in the simulated system following an insertion.

For a deletion move, a random molecule from the simulated system is chosen and translated to a random place within the ideal gas. The probability of selecting the forward move for a deletion is given as:

$$P(\Lambda_\mathrm{p}|x_0) = \frac{1}{2}\frac{1}{N}\frac{d\mathbf{r}}{V_\mathrm{gas}} \tag{6}$$

where the second term gives the probability of selecting, at random, a particle in the simulated system. The reverse protocol for a deletion move is therefore given by:

$$P(\tilde{\Lambda}_\mathrm{p}|\tilde{x}_T) = \frac{1}{2}\frac{1}{(M-N+1)}\frac{d\mathbf{r}}{V_\mathrm{sys}} \tag{7}$$

where $M - N + 1$ is the number of particles in the ideal gas following a deletion.

Secondly, as a predetermined lambda scheme to couple or decouple a molecule is used, the perturbation kernels are therefore deterministic and the cumulative probability of each step in the forward move is equal to that in the reverse move, such that $\alpha(X|\Lambda_\mathrm{p}) = \alpha(\tilde{X}|\tilde{\Lambda}_\mathrm{p})$, and cancels in the acceptance ratio.

Lastly, the ratio of equilibrium probabilities of the two end states, x_0 and $\tilde{x}_T$, after cancellations, is given by the exponent of the total change in energy over the course of an insertion or deletion move:

$$\frac{\pi(\tilde{x}_T)}{\pi(x_0)} = e^{\beta \Delta E(X|\Lambda_\mathrm{p})} \tag{8}$$

Combining the above into Eq. 3 gives a somewhat simplified acceptance ratio for an insertion move:

$$\frac{A(X|\Lambda_{\mathrm{p}})}{A(\tilde{X}|\tilde{\Lambda}_{\mathrm{p}})} = \frac{M-N}{V_{\mathrm{gas}}} \frac{V_{\mathrm{sys}}}{N+1} e^{-\Delta S(X|\Lambda_{\mathrm{p}})} e^{-\beta \Delta E(X|\Lambda_{\mathrm{p}})} \tag{9}$$

and for a deletion move:

$$\frac{A(X|\Lambda_{\mathrm{p}})}{A(\tilde{X}|\tilde{\Lambda}_{\mathrm{p}})} = \frac{N}{V_{\mathrm{sys}}} \frac{V_{\mathrm{gas}}}{(M-N+1)} e^{-\Delta S(X|\Lambda_{\mathrm{p}})} e^{-\beta \Delta E(X|\Lambda_{\mathrm{p}})} \tag{10}$$

We can further simplify Equations 9 and 10 by focusing on just the canonical partition function of the ideal gas reservoir with $M-N$ particles. To simplify the notation, we define G as the number of particles in the gas ($G = M - N$). As this is an ideal gas, the partition function does not have an excess contribution and we can define the ideal component as:

$$Q^{\mathrm{id}}_{\mathrm{GVT}} = \frac{(V_{\mathrm{gas}})^G}{\Lambda^{3G} G!} \tag{11}$$

where G is the number of particles in the ideal gas with volume, V_{gas}, and Λ is the thermodynamic wavelength. It follows that the corresponding ideal Helmholtz free energy can be determined analytically:

$$\begin{aligned} F^{\mathrm{id}}(G) &= -k_{\mathrm{B}} T \ln Q^{\mathrm{id}}_{\mathrm{GVT}} \\ &= -k_{\mathrm{B}} T \ln \left(\frac{(V_{\mathrm{gas}})^G}{\Lambda^{3G} G!} \right) \end{aligned} \tag{12}$$

Assuming an infinitely large ideal gas, we can use Stirling's approximation to remove the factorials in the above equation:

$$F^{\mathrm{id}}(G) \approx -k_{\mathrm{B}} T \left(G \ln \left(\frac{V_{\mathrm{gas}}}{\Lambda^3} \right) - G \ln G + G \right) \tag{13}$$

the derivative of which with respect to the number of particles, leads us to an equation for the ideal chemical potential:

$$\mu^{\mathrm{id}} = \frac{\partial F^{\mathrm{id}}}{\partial G} = -k_{\mathrm{B}} T \ln \left(\frac{V_{\mathrm{gas}}}{G \Lambda^3} \right) \tag{14}$$

which can finally be rewritten in terms of the number density, ρ_{ideal}, for convenience:

$$\mu^{\mathrm{id}} = k_{\mathrm{B}} T \ln(\rho_{\mathrm{ideal}} \Lambda^3) \tag{15}$$

where

$$\rho_{\mathrm{ideal}} = \frac{G}{V} \tag{16}$$

For an infinitely large ideal gas, the ratio of $\frac{M-N}{V_{\text{ideal}}}$ or $\frac{M-N+1}{V_{\text{ideal}}}$ can also be reduced to the number density, ρ_{ideal}, such that Equations 9 (insertion move) and 10 (deletion move) become:

$$\frac{A(X|\Lambda_{\text{p}})}{A(\tilde{X}|\tilde{\Lambda}_{\text{p}})} = \frac{1}{N+1}\frac{V_{\text{sys}}}{\Lambda^3}e^{\beta\mu^{\text{id}}}e^{-\Delta S(X|\Lambda_{\text{p}})}e^{-\beta\Delta E(X|\Lambda_{\text{p}})} \tag{17}$$

and

$$\frac{A(X|\Lambda_{\text{p}})}{A(\tilde{X}|\tilde{\Lambda}_{\text{p}})} = \frac{N\Lambda^3}{V_{\text{sys}}}e^{-\beta\mu^{\text{id}}}e^{-\Delta S(X|\Lambda_{\text{p}})}e^{-\beta\Delta E(X|\Lambda_{\text{p}})} \tag{18}$$

respectively. Where β is equal to $1/k_{\text{B}}T$.

The Adams parameter, for the ideal gas, in its most basic form, can now be introduced as:

$$B = \beta\mu_{\text{gas}} + \ln\left(\frac{V_{\text{sys}}}{\Lambda^3}\right) \tag{19}$$

where μ_{gas} is the chemical potential of the gas reservoir, noting that this is only made up of its ideal component such that $\mu_{\text{gas}} = \mu_{\text{gas}}^{\text{id}}$. By definition, at equilibrium, the chemical potential of the gas is equal to that of the simulated system. We can substitute B into Eqs. 17 and 18 to give:

$$\frac{A(X|\Lambda_{\text{p}})}{A(\tilde{X}|\tilde{\Lambda}_{\text{p}})} = \frac{1}{N+1}e^{B}e^{-\Delta S(X|\Lambda_{\text{p}})}e^{-\beta\Delta E(X|\Lambda_{\text{p}})} \tag{20}$$

and

$$\frac{A(X|\Lambda_{\text{p}})}{A(\tilde{X}|\tilde{\Lambda}_{\text{p}})} = Ne^{-B}e^{-\Delta S(X|\Lambda_{\text{p}})}e^{-\beta\Delta E(X|\Lambda_{\text{p}})} \tag{21}$$

Finally, to further simplify the acceptance ratios we show that if the propagation kernel maintains an equilibrium distribution, such as the BAOAB Langevin integrator[3] used throughout, the conditional path action can be rewritten in terms of the heat change associated with the nonequilibrium move, $q(X|\Lambda_{\text{p}})$:

$$\Delta S(X|\Lambda_{\text{p}}) = -\beta q(X|\Lambda_{\text{p}}) \tag{22}$$

Furthermore, it is also possible to decompose the energy change into the heat released and the nonequilibrium work done, $w(X|\Lambda_{\text{p}})$, during the move:

$$\Delta E(X|\Lambda_{\text{p}}) = w(X|\Lambda_{\text{p}}) + q(X|\Lambda_{\text{p}}) \tag{23}$$

With these two substitutions and subsequent cancellations, the acceptance ratios can be further simplified:
For an insertion:

$$\frac{A(X|\Lambda_{\text{p}})}{A(\tilde{X}|\tilde{\Lambda}_{\text{p}})} = \frac{1}{N+1}e^{B}e^{-\beta w(X|\Lambda_{\text{p}})} \tag{24}$$

and for a deletion:

$$\frac{A(X|\Lambda_{\text{p}})}{A(\tilde{X}|\tilde{\Lambda}_{\text{p}})} = Ne^{-B}e^{-\beta w(X|\Lambda_{\text{p}})} \tag{25}$$

where now the acceptance ratio depends on the work done during the nonequilibrium switch and can be calculated as the sum of the work done on the system at each perturbation step:

$$w(X|\Lambda_{\mathrm{p}}) = \sum_{t=1}^{T} [U(x_t^*) - U(x_{t-1})] \tag{26}$$

where $U(x_t^*)$ is the potential energy of the system at state t before any relaxation and $U(x_{t-1})$ is the potential energy of the system prior to the perturbation, or in other words, the potential energy of relaxed state $t-1$.

1.2 The GCMC Sphere

To further improve the convergence of GCNCMC moves, the user can restrict insertion and deletion moves to a region of particular interest — usually a protein binding site. This prevents moves from occurring in the bulk solvent, which is typically of little interest. In theory, any shape with a calculable volume can be used as the GCMC region, though in practice we use a sphere for its simplicity. Further details on this design consideration can be found in the publication by Samways *et al.*[4]

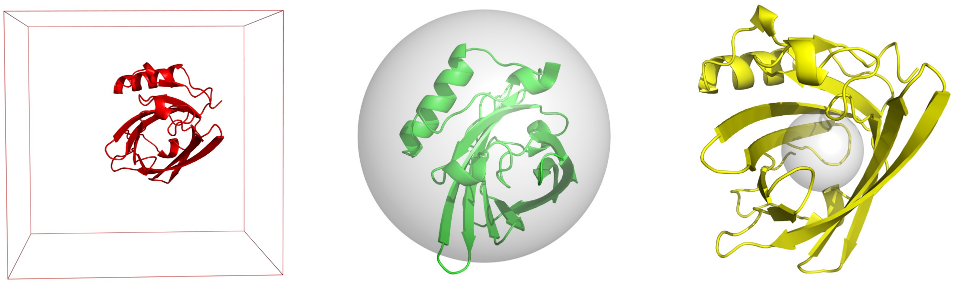

Supplementary Figure 2: **Examples of the GCMC region.** Left: An entire simulation cell. Middle: A sphere that covers all of the protein. Right: A sphere that is centered in a binding site.

There are some additional considerations when the insertions and deletions of particles are attempted only within a subset of the total system volume e.g. a sphere. First, if the molecule being switched lies outside the sphere by the end of the move, the move must be automatically rejected since the reverse process cannot be proposed, breaking the condition for detailed balance. Secondly, the $(N+1)^{-1}$ and N terms in eqs 24 and 25 must be adjusted to account for the fact that the number of molecules in the sphere may change during the nonequilibrium protocol owing to diffusion during the MD propagation steps to give:

$$P_{\mathrm{insert}} = \min\left[1, \frac{1}{N_T} e^{B} e^{-\beta w(X|\Lambda_{\mathrm{p}})}\right] \tag{27}$$

Supplementary Table 1: Atoms used to define the GCMC sphere. The numbering for T4L99A and MUP1 corresponds to that of the original PDB (T4L99A: 4w51 MUP1: 1i06). Host guest numbering as per the input file provided. Where multiple atoms are present the center of the sphere is an average of all atoms (center of geometry).

System	Atom(s)		Radius / Å
Host Guest	C34	C7	5.0
T4L99A (full protein)	Phe104-CA	Glu11-CA	26.5
T4L99A (binding site)	Leu84-CA	Ala99-CA	8.0
MUP1 (full protein)	Gly118-CA		22.0
MUP1 (binding site)	Phe56-CA	Leu105-CA	5.5

$$P_{\text{delete}} = \min\left[1, N_0 e^{-B} e^{-\beta w(X|\Lambda_{\text{p}})}\right] \tag{28}$$

where N_0 is the number of particles in the GCMC sphere in the initial state and N_T is the corresponding number for the proposed state.

Implementation-wise, the sphere, with radius r, is constructed in two ways. One, the user can anchor the center of the sphere to a protein atom or, to the center of geometry of multiple protein atoms. This is the approach adopted in this work as the sphere will then follow the protein as the simulation propagates. Alternatively, the sphere can be defined using Cartesian coordinates where it will remain fixed during the simulation. The sphere details for the systems studied in this work can be found in Table 1.

1.3 Adams Value and Equilibrium with a Reference Solution

In the previous sections, the Adams value, B, was defined as:

$$B = \beta\mu_{\text{gas}} + \ln\left(\frac{V_{\text{sys}}}{\Lambda^3}\right) \qquad \text{(19 revisited)}$$

where μ_{gas} is the chemical potential of the ideal gas, V_{sys} is the volume of the region in which insertion and deletion moves are attempted (also denoted as V_{GCMC}), and Λ is the thermodynamic wavelength of the particle.

The theoretical definitions require that the system, or GCMC region, be in equilibrium with an ideal gas. It follows, that this ideal gas can also be in equilibrium with an aqueous solution and by coupling these equilibria, it means we can effectively set the chemical potential of the ideal gas to that of some arbitrary reference solution, such as a bulk solvent or a mixture to observe a more physically meaningful equilibrium.[5] Supplementary Figure 3 shows this graphically.

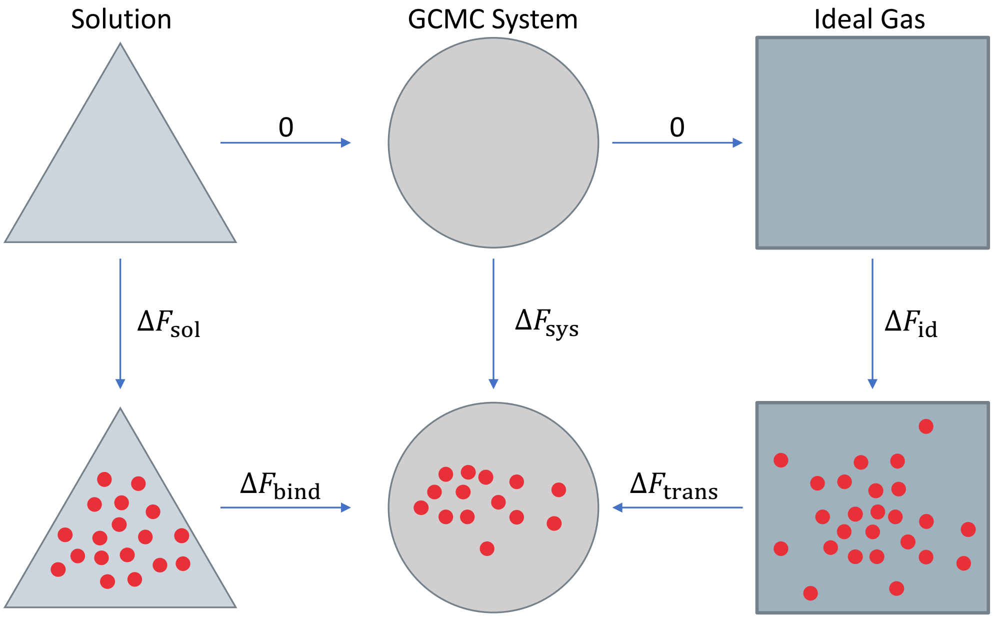

Supplementary Figure 3: Thermodynamic cycle linking the binding of molecules from solution to the GCMC system with the binding of molecules from the ideal gas. The left triangles represent a solution phase, the circles represent the GCMC region/system and rectangles are the ideal gas. The top row indicates fully empty systems while the bottom row contains some particles indicated by the red dots.

Supplementary Figure 3 shows that our simulated system is now in equilibrium with a reference solution (via a gas reservoir) which is generally more useful when considering binding from solvent. It follows, that the chemical potential of the reference solution now has both an excess and ideal component such that:

$$\mu_{\mathrm{sol}} = \mu^{\mathrm{id}} + \mu'_{\mathrm{sol}} \tag{29}$$

where μ'_{sol} is the excess chemical potential of a molecule in the reference solution. The ideal chemical potential is again defined as:

$$\mu^{\mathrm{id}} = k_{\mathrm{B}} T \ln \frac{N \Lambda^3}{V} \tag{30}$$

where N is the number of particles in the reference, Λ is the thermodynamic wavelength, and V is the volume of the reference solution. Under standard state conditions, the number density, N/V is well defined as $1/V^{\circ}$ giving:

$$\mu = k_{\mathrm{B}} T \ln \frac{\Lambda^3}{V^{\circ}} + \mu'_{\mathrm{sol}} \tag{31}$$

Substituting this into the equation for the Adams value, B, the standard state Adams value in equilibrium with an arbitrary solution can be defined as:

$$\begin{aligned} B_{\text{eq}}^{\ominus} &= \beta\left(\mu'_{\text{sol}} + k_{\text{B}}T\ln\left(\frac{\Lambda^3}{V^{\ominus}}\right)\right) + \ln\left(\frac{V_{\text{sys}}}{\Lambda^3}\right) \\ &= \beta\mu'_{\text{sol}} + \ln\left(\frac{V_{\text{GCMC}}}{V^{\ominus}}\right) \end{aligned} \tag{32}$$

where the Adams value now depends on the excess chemical potential of the molecule of interest in a reference solution. Note, that we have changed the notation of V_{sys} to V_{GCMC} as the GCMC region may not be the whole system (e.g. a sphere). An alternate derivation can be found in the publication by Ross *et al.*[5]

The standard states for water and small molecules are well-defined as 55 M and 1 M, respectively. However, in many cases, simulating a molecule, such as a fragment, at a concentration that is not the standard state is more experimentally relevant. For example, fragment-like molecules tend to bind to their targets in the micromolar to millimolar range. In such situations where the molecule in a reference solution (the solution with which our simulated system is in equilibrium) deviates from the standard state concentration, we define the Adams value with a specific concentration dependence to reflect equilibrium with a solution of concentration, c:

$$B_{\text{eq}}(c) = \beta\mu'_{\text{sol}} + \ln\left(\frac{V_{\text{GCMC}}}{V(c)}\right) \tag{33}$$

where $V(c)$ is now the average volume occupied by a molecule at concentration, c, as can be trivially calculated by:

$$V(c) = \frac{1}{N_{\text{A}}c} \tag{34}$$

1.4 Excess Chemical Potential

A pre-requisite for any GCNCMC simulation is the calculation of the excess chemical potential, μ'_{sol}, of the molecule of interest in a reference solution with which the GCMC region is in equilibrium. In other words, how favorable that molecule is in a given solution will ultimately affect the equilibrium between a binding site and the solution. Conceptually, a hydrophobic molecule in water is likely to be 'unhappy' meaning that it would be easier to insert and harder to delete from a hydrophobic protein region.

As mentioned in the main text, the value of the excess chemical potential is dependent on all the constituents of the reference solution and is approximated as:

$$\mu'_{\text{sol}} \approx \frac{\Delta F'}{\Delta N} \tag{35}$$

where $\Delta F'$ is the change in the free energy associated with a change in the number of molecules, ΔN. By taking the smallest possible value of ΔN as 1, then the excess chemical potential can be calculated as:

$$\mu'_{\text{sol}} = F'(N+1) - F'(N) \tag{36}$$

where $F'(N)$ is the excess free energy of a system with N molecules and $F'(N+1)$ is for a system with a molecule added.

In this work, except for concentration simulations, we have approximated the excess chemical potential of a particular molecule to be equal to the hydration free energy of that molecule at infinite dilution. In previous works using water[1, 4, 5], this approximation holds because we are interested in sampling the relationship between binding sites and bulk water, where the excess chemical potential of water in water is indeed its hydration free energy. Here, we are more interested in sampling the equilibrium between a non-water molecule in a binding site and an arbitrary concentration of the same non-water molecule in water. This complicates the parameterization of the excess chemical potential as we now have a multi-component mixture rather than a bulk solution.

Therefore, the excess chemical potential of a molecule in solution is equivalent to the solvation free energy of that molecule in that solution. As such, it will depend on the number of molecules of the same kind already in the solution (assuming a 2-component mixture). This dependence is something often neglected by traditional hydration/solvation free energy calculations which are often performed at “infinite dilution” whereby a single molecule is coupled/decoupled from a box containing only water. At sufficiently low concentrations, such as those at which molecules bind to proteins, this approximation holds particularly well since the probability of interacting with another molecule of the same kind is low and thus acts as if it is in water alone. Furthermore, calculating the excess chemical potential for very low concentrations is difficult in practice, as large simulation boxes are required to achieve such a low concentration of molecules.

At higher concentrations, the probability of a single molecule interacting with another of its kind is greater and thus contributes to the excess chemical potential. However, the effects of these interactions are complex, hard to predict, and differ greatly between molecules. As an example, the free energy of adding an apolar benzene molecule to a box already containing N molecules of benzene would be more favorable than adding to a pure box of polar water.

To investigate the effect of higher concentrations on excess chemical potential, we take inspiration from a publication by Ross *et al.*[6] who calibrated the chemical potential of exchanging water molecules with salt pairs for an increasing number of salt pairs already in the system. Here, we use the insertion and deletion functionality of our code to sequentially add acetone molecules to a box of 887 TIP3P waters up to a maximum of 20 molecules before cycling back to zero. We repeat this cycle 250 times recording the works of each addition/removal of acetone. These nonequilibrium works can then be used to calculate an equilibrium free energy using the Bennett Acceptance Ratio[7] to calculate the free energy of adding a molecule to the system as a function of N.

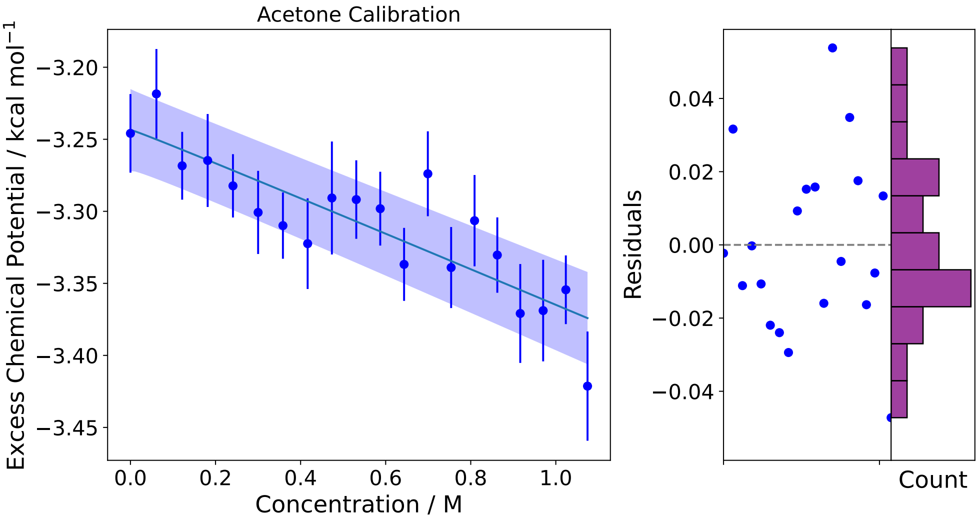

Supplementary Figure 4: **Excess chemical potential of Acetone as a function of concentration.** The data was fitted to a function of the form $y = a + b\sqrt{x} + dx$. Error bars are the standard error of the mean value. The shaded region corresponds to the standard error of the fits.

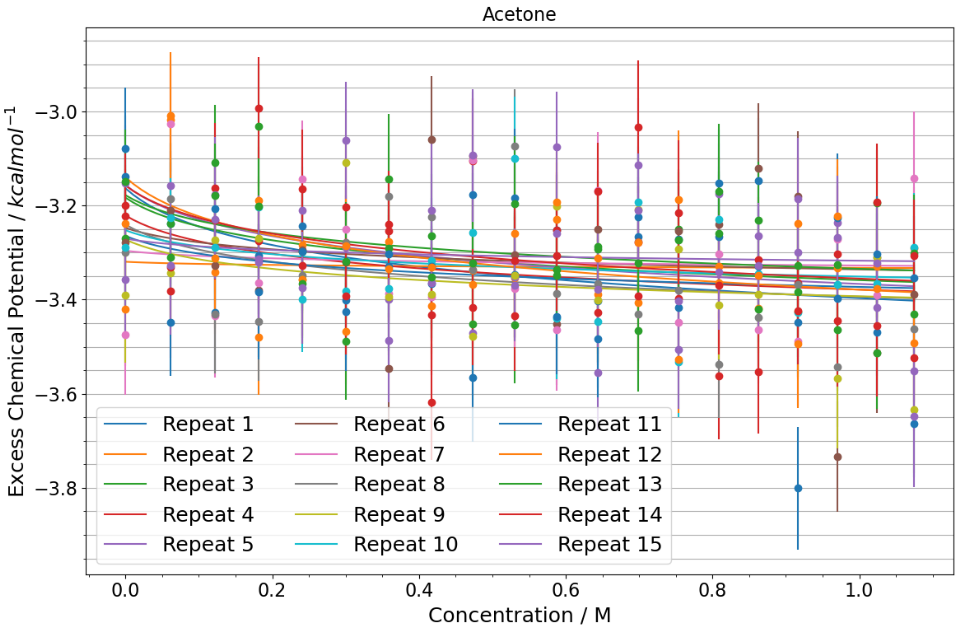

Supplementary Figure 5: **Excess chemical potential of Acetone as a function of concentration for 15 independent repeats.** The error bars represent the uncertainty estimation for the BAR estimator in pymbar.

Fitting a curve to this function means that, in principle, the excess chemical potential for any concentration could be found, though in practice would require many simulations at a range of concentrations. What is clear from Supplementary Figure 4 is that the excess chemical potential is indeed dependent on the concentration, though, at least for acetone, this dependence is small and within the range of approximately 0.2-0.3 kcal mol^{-1}. Typically, comparing free energies with such a low dynamic range is not recommended, as random noise in the data can hamper performance. Supplementary Figure 5 shows the results from 15 independent repeats on the same plot to stress this element of random noise. Further, in a traditional protein-ligand binding free energy calculation, one would generally not worry about such a small difference but as we have seen previously, small differences can have an impact on the overall concentration of the system.[4]

We note that this sort of calibration can be expensive and therefore not viable for a large data set of molecules. As such, in this work, excluding the bulk concentration simulations (Main Text Sec. 2.3) where we simulate at higher concentrations, we assume the excess chemical potential value to be equal to the infinitely dilute hydration free energy, as this is sufficient for the concentrations (nM-mM) simulated in the protein-ligand applications.

For validation, the infinitely dilute excess chemical potentials of the molecules studied in this paper, where data are available, are plotted against experiment and calculated hydration free energies as reported in the FreeSolv database (Supplementary Figure 6). Actual values are reported in the individual sections of this SI.

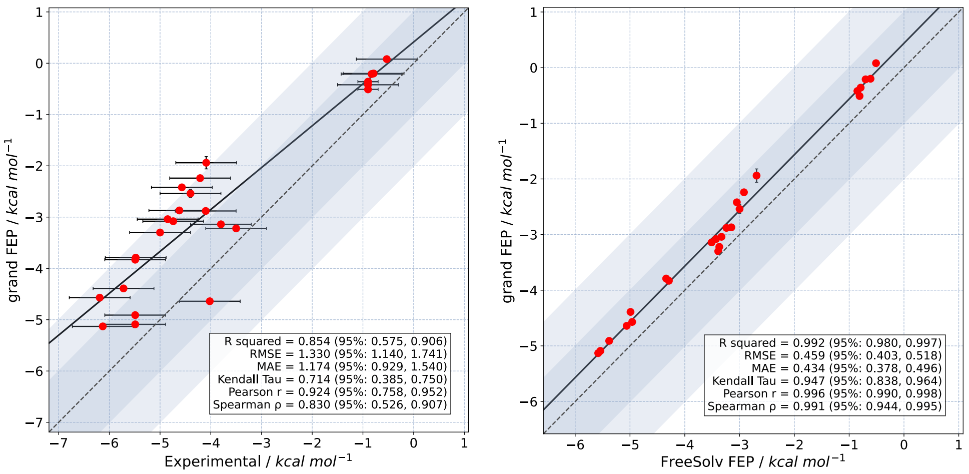

Supplementary Figure 6: Calculated excess chemical potentials of ligands studied in this paper (hydration free energies) vs. experiment and previously published FEP results in the FreeSolv database (where data available).

1.5 Linking B_{50} to ΔF_{trans}

Throughout the main manuscript, we have stated that B_{50} is equal to the dimensionless free energy of transfer ($\beta \Delta F_{\text{trans}}$). To show this we refer back to the thermodynamic cycle in Supplementary Figure 3. This proof also shows why K_{D} can be directly calculated from B_{50}.

First, from the thermodynamic cycle, we state that:

$$\Delta F_{\text{trans}} = -\Delta F_{\text{id}} + \Delta F^{\ominus}_{\text{sol}} + \Delta F^{\ominus}_{\text{bind}} \tag{37}$$

where ΔF_{trans} is the free energy of transferring a molecule from the ideal gas into the GCMC system. ΔF_{id} is the free energy difference associated with changing the number of molecules in the ideal gas, $\Delta F^{\ominus}_{\text{sol}}$ is the free energy change associated with adding molecules to a solution and $\Delta F^{\ominus}_{\text{bind}}$ is the binding free energy.

The free energy of changing the number of particles in the ideal gas, ΔF_{id} can be calculated analytically[5]:

$$\Delta F_{\mathrm{id}}(N_{\mathrm{i}} \to N_{\mathrm{f}}) = k_{\mathrm{B}}T \ln\left(\frac{N_{\mathrm{f}}!}{N_{\mathrm{i}}!}\right) - k_{\mathrm{B}}T(N_{\mathrm{f}} - N_{\mathrm{i}}) \ln\left(\frac{V_{\mathrm{ideal}}}{\Lambda^3}\right) \tag{38}$$

The free energy associated with adding a particle to a solution, $\Delta F^{\ominus}_{\mathrm{sol}}$, also the solvation free energy, is given by the the chemical potential:

$$\begin{aligned} \Delta F^{\ominus}_{\mathrm{sol}}(N_{\mathrm{i}} \to N_{\mathrm{f}}) &= (N_{\mathrm{f}} - N_{\mathrm{i}})\mu_{\mathrm{sol}} \\ &= (N_{\mathrm{f}} - N_{\mathrm{i}})(\mu'_{\mathrm{sol}} + k_{\mathrm{B}}T \ln(\rho_{\mathrm{sol}}\Lambda^3)) \end{aligned} \tag{39}$$

where ρ_{sol} is the number density given by N/V. Under standard state conditions, the number density is well defined as $1/V^{\ominus}$ such that:

$$\Delta F^{\ominus}_{\mathrm{sol}}(N_{\mathrm{i}} \to N_{\mathrm{f}}) = (N_{\mathrm{f}} - N_{\mathrm{i}})\left(\mu'_{\mathrm{sol}} + k_{\mathrm{B}}T \ln\left(\frac{\Lambda^3}{V^{\ominus}}\right)\right) \tag{40}$$

And finally, the binding free energy, $\Delta F^{\ominus}_{\mathrm{bind}}$, is, as explained previously, given by:

$$\Delta F^{\ominus}_{\mathrm{bind}} = k_{\mathrm{B}}T \ln(K_{\mathrm{D}}/c^{\ominus}) \tag{41}$$

where $c^{\ominus}$ can be given by:

$$c^{\ominus} = \frac{1}{N_{\mathrm{A}}V^{\ominus}} \tag{42}$$

Combining the three results gives an equation for the transfer free energy:

$$\begin{aligned} \beta\Delta F_{\mathrm{trans}}\ (N_{\mathrm{i}} \to N_{\mathrm{f}}) = -\ln\left(\frac{N_{\mathrm{f}}!}{N_{\mathrm{i}}!}\right) &+ (N_{\mathrm{f}} - N_{\mathrm{i}}) \ln\left(\frac{V_{\mathrm{ideal}}}{\Lambda^3}\right) \\ +(N_{\mathrm{f}} - N_{\mathrm{i}}) &\left(\mu'_{\mathrm{sol}} + k_{\mathrm{B}}T \ln\left(\frac{\Lambda^3}{V^{\ominus}}\right)\right) \\ &+ \ln(K_{\mathrm{D}}/c^{\ominus}) \end{aligned} \tag{43}$$

Evaluating each term of the above equation for $N_{\mathrm{i}} = 0$ and $N_{\mathrm{f}} = 1$, and simplifying, we get:

$$\begin{aligned} \beta\Delta F_{\mathrm{trans}} &= \ln\left(\frac{V_{\mathrm{ideal}}}{\Lambda^3}\right) + \mu'_{\mathrm{sol}} + k_{\mathrm{B}}T \ln\left(\frac{\Lambda^3}{V^{\ominus}}\right) + \ln(K_{\mathrm{D}}/c^{\ominus}) \\ &= \mu'_{\mathrm{sol}} + k_{\mathrm{B}}T \ln\left(\frac{V_{\mathrm{ideal}}K_{\mathrm{D}}N_{\mathrm{A}}V^{\ominus}}{V^{\ominus}}\right) \\ \beta\Delta F_{\mathrm{trans}} &= \beta\mu'_{\mathrm{sol}} + \ln(V_{\mathrm{sys}}K_{\mathrm{D}}N_{\mathrm{A}}) = B_{50} \end{aligned} \tag{44}$$

where the final result of Equation 44 is equal to the value of B_{50} given in Equation 51. Note that for this derivation, we assume that the volume of the ideal gas and the volume of the system are equivalent in terms of their ideal component as in Ross *et al.*[8]

1.6 Equivalence of GCI to Logistic Function

In previous work, we have calculated the standard state binding free energies of water molecules using grand canonical integration (GCI).[5, 8] The GCI equation is built upon the thermodynamic cycle presented in Supplementary Figure 3 and requires integrating over a range of B values:

$$\beta\Delta G^{\ominus}_{\mathrm{gci}}\left(N_{\mathrm{i}} \rightarrow N_{\mathrm{f}}\right) = N_{\mathrm{f}}B_{\mathrm{f}} - N_{\mathrm{i}}B_{\mathrm{i}} - (N_{\mathrm{f}} - N_{\mathrm{i}})\left[\beta\mu'_{\mathrm{sol}} + \ln\left(\frac{V_{\mathrm{GCMC}}}{V^{\ominus}}\right)\right] - \int_{B_{\mathrm{i}}}^{B_{\mathrm{f}}} N(B)\,\mathrm{d}B \tag{45}$$

where B_k is the Adams parameter for which there is an average of N_k molecules, V_{GCMC} is the volume of the GCMC region, $V^{\ominus}$ is the standard state volume, and μ'_{sol} is the excess chemical potential of the particle.

This GCI equation can be applied in the same way to small molecule binding. For the most common case of one molecule binding we find that:

$$\beta\Delta G^{\ominus}_{\mathrm{gci}}\left(0 \rightarrow 1\right) = B_{\mathrm{f}} - \left[\beta\mu'_{\mathrm{sol}} + \ln\left(\frac{V_{\mathrm{GCMC}}}{V^{\ominus}}\right)\right] - \int_{B_{\mathrm{i}}}^{B_{\mathrm{f}}} N(B)\,\mathrm{d}B \tag{46}$$

Previous studies[8] also showed that for a binding site that can only bind one molecule, a logistic function with respect to the Adams value can be fitted such that:

$$N(B_{\mathrm{eq}}) = \frac{1}{1 + \exp(B_{50} - B_{\mathrm{eq}})} \tag{47}$$

where B_{eq} is the equilibrium Adams value and B_{50} is the Adams value required to return a 50% bound occupancy. The value of B_{50} is equal to the dimensionless free energy of transfer from gas phase to the binding site, $\beta\Delta F_{\mathrm{trans}}$. It follows, that given the concentration dependence in B, a similar function can be applied to concentration. Note, that we typically simulate concentrations on a log scale.

$$N(\log_{10}(c)) = \frac{1}{1 + \exp(\log_{10}(K_{\mathrm{D}}) - \log_{10}(c))} \tag{48}$$

where the value for B_{50} has now be replaced by $\log_{10} K_{\mathrm{D}}$, the concentration required to maintain a 50% bound occupancy. In practice, one could fit the sigmoid curve with respect to B and convert to concentration using the following relationship:

$$B_{\mathrm{eq}} = \beta\mu'_{\mathrm{sol}} + \ln(N_{\mathrm{A}} c_{\mathrm{L}} V_{\mathrm{GCMC}}) \tag{49}$$

where

$$c_{\mathrm{L}} = \frac{1}{N_{\mathrm{A}} V(c)} \tag{50}$$

where N_{A} is Avogadro's constant and $V(c)$ is the volume a molecule occupies at concentration, c. At a standard state of $1M$, $V(1) = V^{\ominus} = 1.661 \times 10^{-24} dm^{-3}$. The corresponding concentration at B_{50} is K_{D}, the dissociation constant.

$$B_{50} = \beta\mu'_{\mathrm{sol}} + \ln(N_{\mathrm{A}} K_{\mathrm{D}} V_{\mathrm{GCMC}}) \tag{51}$$

Substituting the logistic function (Eq. 47) into the GCI equation (Eq. 46) we find:

$$\beta\Delta G^{\ominus}_{\text{gci}}\ (0 \rightarrow 1) = B_{\text{f}} - \left[\beta\mu'_{\text{sol}} + \ln\left(\frac{V_{\text{GCMC}}}{V^{\ominus}}\right)\right] - \int_{B_{\text{i}}}^{B_{\text{f}}} \frac{1}{1+\exp(B_{50}-B_{\text{eq}})}\text{d}B \quad (52)$$

The integral can now be evaluated analytically such that:

$$\begin{aligned}\int_{B_{\text{i}}}^{B_{\text{f}}} N(B)\text{d}B &= \int_{B_{\text{i}}}^{B_{\text{f}}} \frac{1}{1+\exp(B_{50}-B_{\text{eq}})}\text{d}B \\ &= \int_{B_{\text{i}}}^{B_{\text{f}}} \frac{\exp(B_{\text{eq}}-B_{50})}{1+\exp(B_{\text{eq}}-B_{50})}\text{d}B\end{aligned} \quad (53)$$

and by using the following relationship:

$$\frac{\text{d}}{\text{d}x}\ln(1+\exp(x)) = \frac{\exp(x)}{1+\exp(x)} \quad (54)$$

the integral can be evaluated analytically as:

$$\begin{aligned}\int_{B_{\text{i}}}^{B_{\text{f}}} N(B)\text{d}B &= \ln[1+\exp(B-B_{50})]\ |_{B_{\text{i}}}^{B_{\text{f}}} \\ &= \ln\left[\frac{1+\exp(B_{\text{f}}-B_{50})}{1+\exp(B_{\text{i}}-B_{50})}\right]\end{aligned} \quad (55)$$

For the logistic equation to be valid between 0 and 1, the concentrations, and therefore B values, must be chosen such that $N(B_{\text{i}}) = 0$ and $N(B_{\text{f}}) = 1$. We therefore require that $B_{\text{i}} - B_{50} \ll 0$ and $B_{\text{f}} - B_{50} \gg 0$.
When $(B_{\text{i}} - B_{50}) \rightarrow -\infty$ we get:

$$1+\exp(B_{\text{i}}-B_{50}) = 1 \quad (56)$$

and as $(B_{\text{f}} - B_{50}) \rightarrow \infty$ we get:

$$1+\exp(B_{\text{f}}-B_{50}) = \exp(B_{\text{f}}-B_{50}) \quad (57)$$

Putting these two limits into Eq. 53 shows that:

$$\int_{B_{\text{i}}}^{B_{\text{f}}} N(B)\text{d}B = B_{\text{f}} - B_{50} \quad (58)$$

Substituting this result into Eq. 46 we get:

$$\beta\Delta G^{\ominus}_{\text{gci}} = B_{\text{f}} - \left[\beta\mu'_{\text{sol}} + \ln\left(\frac{V_{\text{GCMC}}}{V^{\ominus}}\right)\right] - B_{\text{f}} - B_{50} \quad (59)$$

Simplifying and substituting in Eq. 51 for B_{50} we get:

$$\begin{aligned}\beta\Delta G^{\ominus}_{\mathrm{gci}} &= -\beta\mu'_{\mathrm{sol}} - \ln\left(\frac{V_{\mathrm{GCMC}}}{V^{\ominus}}\right) + B_{50} \\ &= -\beta\mu'_{\mathrm{sol}} - \ln\left(\frac{V_{\mathrm{GCMC}}}{V^{\ominus}}\right) + \beta\mu'_{\mathrm{sol}} + \ln(N_{\mathrm{A}}K_{\mathrm{D}}V_{\mathrm{GCMC}}) \\ &= \ln(N_{\mathrm{A}}K_{\mathrm{D}}V_{\mathrm{GCMC}}) - \ln\left(\frac{V_{\mathrm{GCMC}}}{V^{\ominus}}\right) \\ &= \ln(N_{\mathrm{A}}K_{\mathrm{D}}V^{\ominus})\end{aligned} \tag{60}$$

given $N_{\mathrm{A}}V^{\ominus} = 1M^{-1}$ we finally arrive at a well known equation showing that binding affinity can be calculated either via the GCI equation or from B_{50} directly.

$$\begin{aligned}\beta\Delta G^{\ominus}_{\mathrm{gci}} &= \ln(N_{\mathrm{A}}K_{\mathrm{D}}V^{\ominus}) \\ \Delta G^{\ominus}_{\mathrm{gci}} &= \beta^{-1}\ln(K_{\mathrm{D}}/c^{\ominus}) \\ \Delta G^{\ominus}_{\mathrm{gci}} &= \Delta G^{\ominus}_{\mathrm{log}}\end{aligned} \tag{61}$$

2 Supplementary Discussion and Figures

2.1 Bulk Concentration Simulations

To fully control the concentration of the system we must not only perform ligand GCNCMC moves but also water moves are needed to maintain the balance between the two species. For example, if we start from a pure water box at the correct density, we would need to be able to delete water molecules to make space for the ligand molecules. In other words, without water moves, the maximal concentration achievable by the ligand is limited by the size of the box. As such, we require a rigorous parameterization for the excess chemical potential of both the small molecule and water, $\mu'_{\mathrm{sol,L}}$ & $\mu'_{\mathrm{sol,W}}$. We find that for both acetone and pyrimidine, the value of $\mu'_{\mathrm{sol,W}}$ does not differ from that of bulk water as it is still the dominant species in the solution and is close to bulk concentration. The overall results are presented in Table 2 and the main text.

It has been shown previously that sampling concentrations via GCMC and GCNCMC moves can be very sensitive to the parametrization of the excess chemical potential, and the large values of N and V_{GCMC}, greatly magnify any errors in the calculated parameters.[1, 4, 6, 9] We refer the reader to a publication by Ross *et al.* for a deeper understanding of this issue of sensitivity and fluctuations.[6] Note that for typical protein-ligand applications, this sensitivity becomes less of an issue as there are fewer interacting GCMC molecules, the volume of the GCMC region is smaller, and simulations are performed at more dilute concentrations where the difference in μ'_{sol} values becomes negligible.

To illustrate this point we fixed the excess chemical potential of acetone to its hydration free energy (-3.17 kcal mol^{-1}) and performed simulations starting from a pure water box setting the desired concentration to 0.1 M, 0.5 M, and, 1.0 M. These results are shown in Supplementary Figure 7. The results show that as we aim for

higher concentrations using the same μ'_{sol} value we find a greater over estimation of the concentration. This makes sense when we consider the equilibrium between the reference solution and the simulated system and the role of the excess chemical potential. At 1 M, the excess chemical potential of acetone in water (-3.31 kcal mol^{-1}) is more negative than what is predicted at infinite dilution owing to favorable interactions with other acetone molecules. By using the infinitely dilute value, the equilibrium is being shifted to the simulated system driving insertion moves away from the less favorable reference solution into the more favorable, higher concentration, simulated system.

In line with our previous statements, the overestimation becomes less pronounced at lower concentrations, with 0.5 M ($\mu'_{\text{sol}} = -3.25$ kcal mol^{-1}) having been slightly over estimated but with 0.1 M being well produced where the excess chemical potential is 3.21 kcal mol^{-1}. It is clear that at lower concentrations where the value of the excess chemical potential tends towards the infinitely dilute value, the concentration is well reproduced.

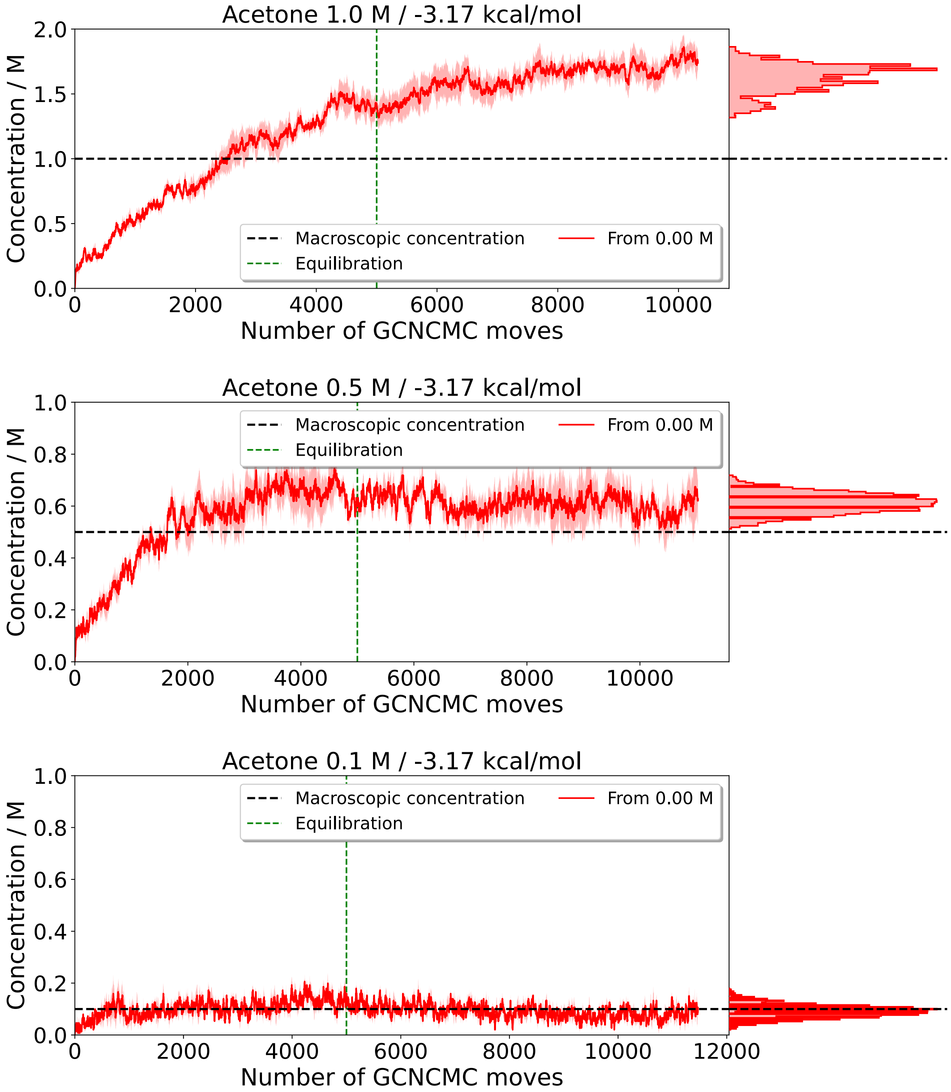

Supplementary Figure 7: **Acetone concentration as a function of time with a fixed chemical potential.** Top: 1.0 M, Middle: 0.5 M, Bottom: 0.1 M.

Supplementary Table 2: Simulation parameters, starting concentrations and final results for the bulk concentration simulations of acetone and pyrimidine. Quoted errors represent one standard error on the mean.

Ligand (Initial conc.)	Ace. (0 M)	Ace. (0.93 M)	Pyr. (0 M)	Pyr. (0.47 M)
$\mu'_{\text{sol,L}}$ / kcal mol^{-1}	-3.25 ± 0.03		-4.49 ± 0.02	
$V_L(c_\text{L})$ / Å^3	3360 ± 0.9		16312 ± 4	
$\mu'_{\text{sol,W}}$ / kcal mol^{-1}	-6.09 ± 0.01		-6.09 ± 0.01	
$V_W(c_W)$ / Å^3	31.5 ± 0.01		30.6 ± 0.01	
Desired [L] / M	0.49		0.1	
Average [L] / M	0.55 ± 0.02	0.56 ± 0.02	0.10 ± 0.01	0.10 ± 0.01
Median [L] / M	0.53	0.55	0.11	0.10

2.2 β-cyclodextrin

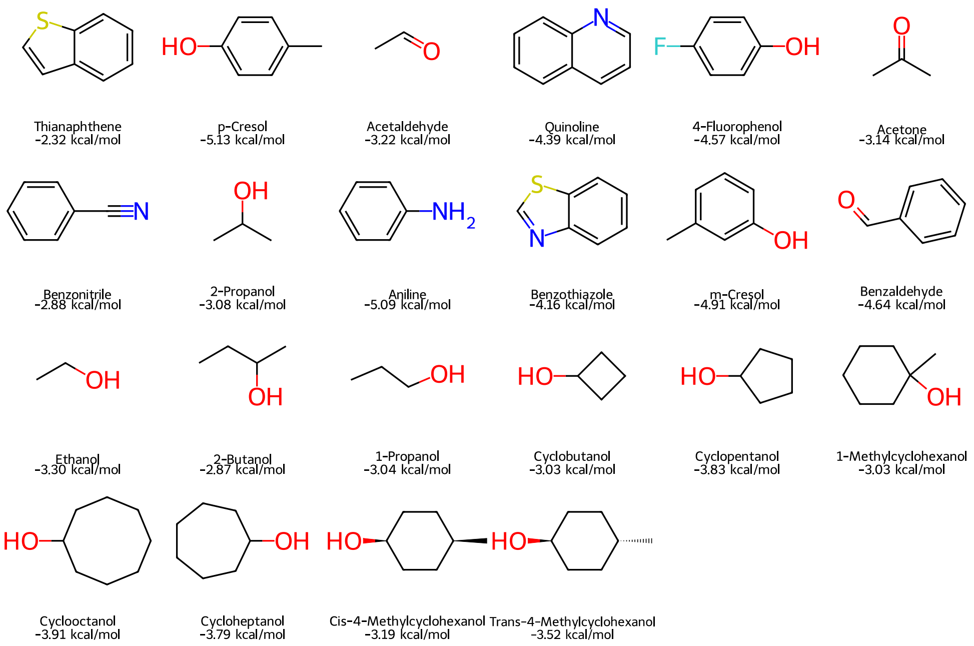

Supplementary Figure 8: Guest ligands for the binding to β-cyclodextrin and their calculated values of excess chemical potential, μ'. Error bars are on the order of 0.01 kcal mol^{-1} and can be found in the Supplementary Data.

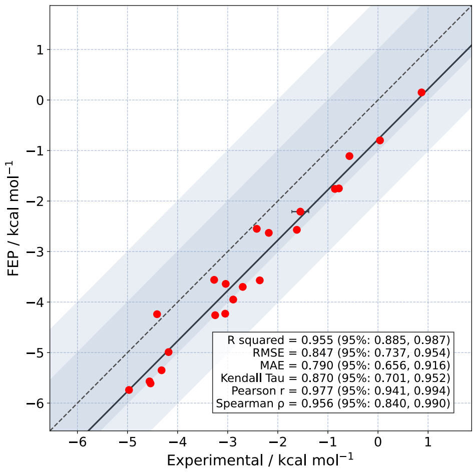

Supplementary Figure 9: **Calculated $\Delta G^{\ominus}$ from FEP calculations vs. experimental data for β-cyclodextrin.** The error on the ABFE results are the standard error of the mean of 4 individual repeats.

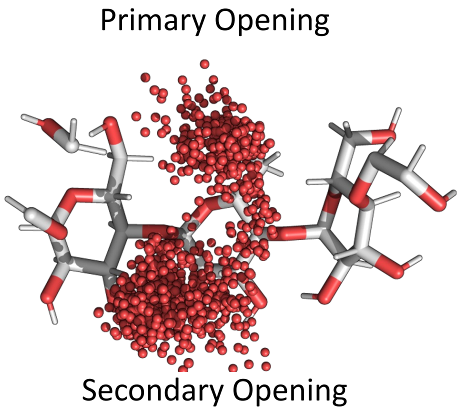

Supplementary Figure 10: **Overlaid frames from GCNCMC simulations of *para*-cresol binding to β-cyclodextrin.** GCNCMC simulations show a preference for the polar group of the p-cresol guest (red spheres) to point out the wider secondary opening. Note, that the depiction of the host is that of the first frame only.

2.3 T4L99A

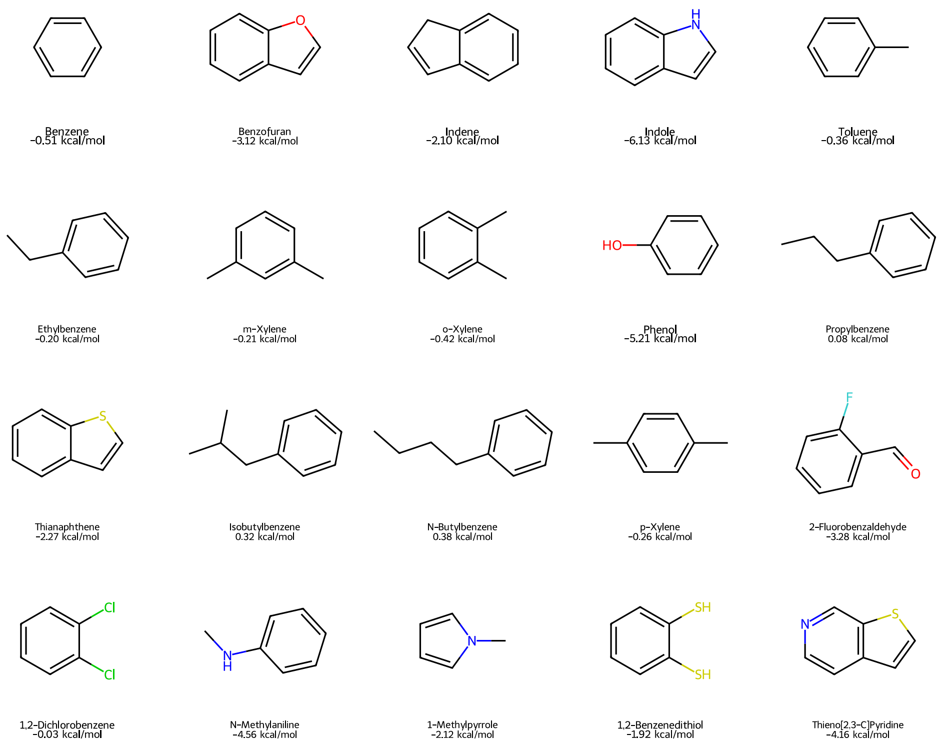

Supplementary Figure 11: T4L99A ligands and their calculated values of excess chemical potential, μ'. Error bars are on the order of 0.02 kcal mol^{-1} and can be found in the Supplementary Data.

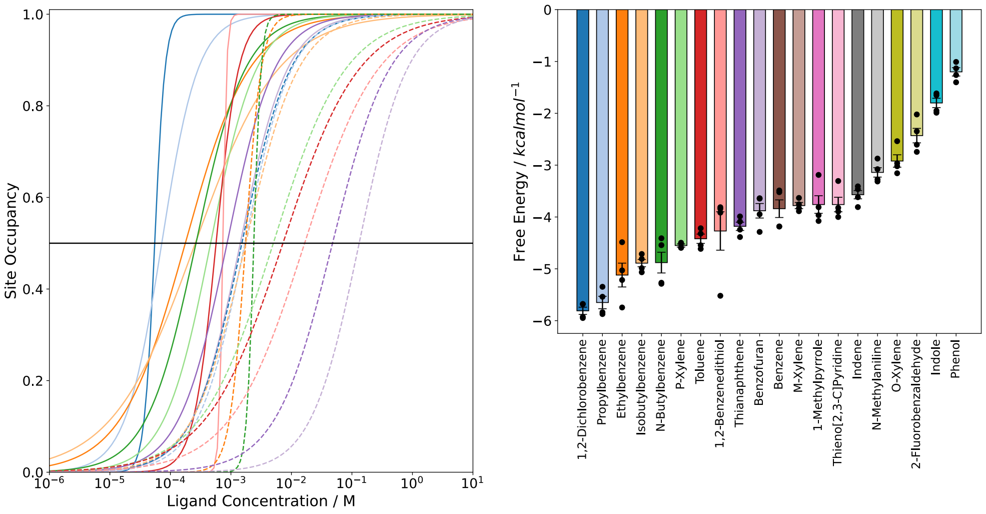

Supplementary Figure 12: **Titration curves for ligands binding to T4L99A.** Calculated free energies are derived from the ligand concentration which gives 50% bound occupancy (K_D). The error is the standard error of the mean of four repeats.

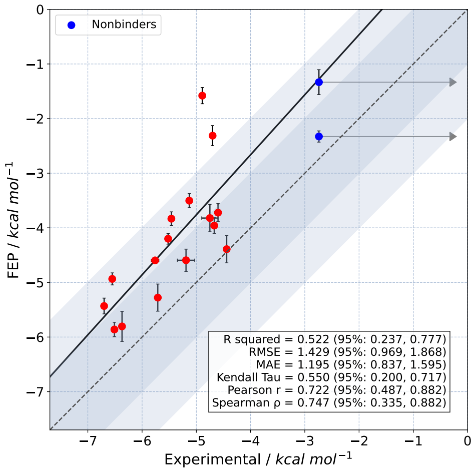

Supplementary Figure 13: **Calculated $\Delta G^{\ominus}$ from FEP calculations vs. experimental data for T4L99A.** The error on the ABFE results are the standard error of the mean of 4 individual repeats.

2.3.1 Multiple Binding Modes in Titrations

Below are examples of where titration calculations sample the multiple binding modes of various T4 ligands.

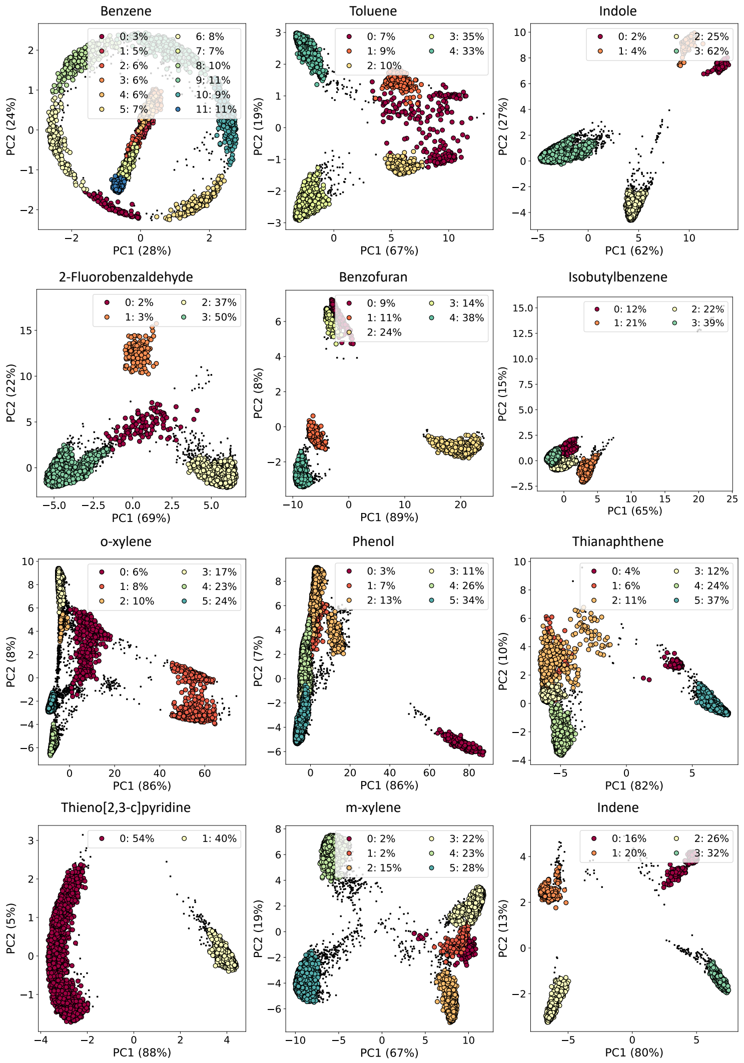

Supplementary Figure 14: **Compilation of all the binding modes sampled in GCNCMC titrations.** Frames from titration simulations at B values returning an average occupancy between 0.4 and 0.6 were clustered using CLoNe[10] based on their pairwise RMSD and projected into PCA space. Populations of each cluster are provided in the legend.

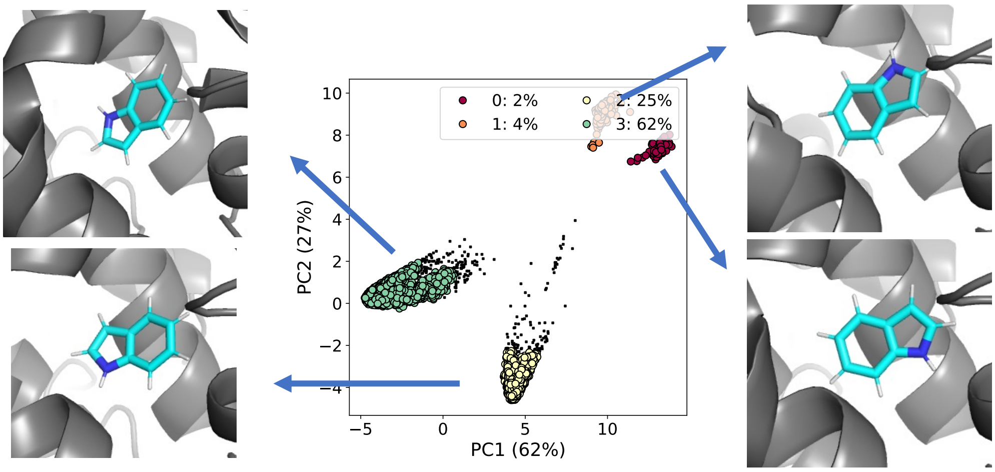

Supplementary Figure 15: Four binding modes of indole sampled within GCNCMC simulations

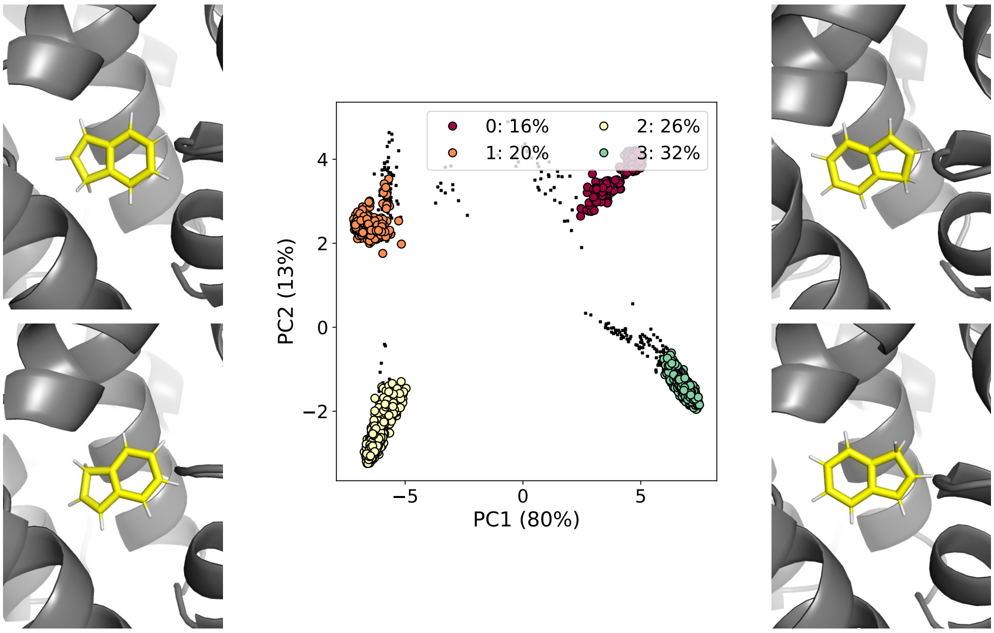

Supplementary Figure 16: Four binding modes of indene sampled within GCNCMC simulations

2.3.2 Titration Convergence Studies

To assess the convergence in our titration calculations, we segment the data based on the number of GCNCMC moves performed and evaluate the free energy estimate by plotting titration curves as if only that number of moves were performed. Representative plots (best, worst, and average) are shown below (Supplementary Figure 17) with the remainder provided in the supplementary data (https://github.com/essex-lab/grandlig-paper). On average, GCNCMC titrations converge within 700 moves (46% of moves). Generally, the worst-performing titrations are those where few B values are simulated in the vicinity of B_{50}.

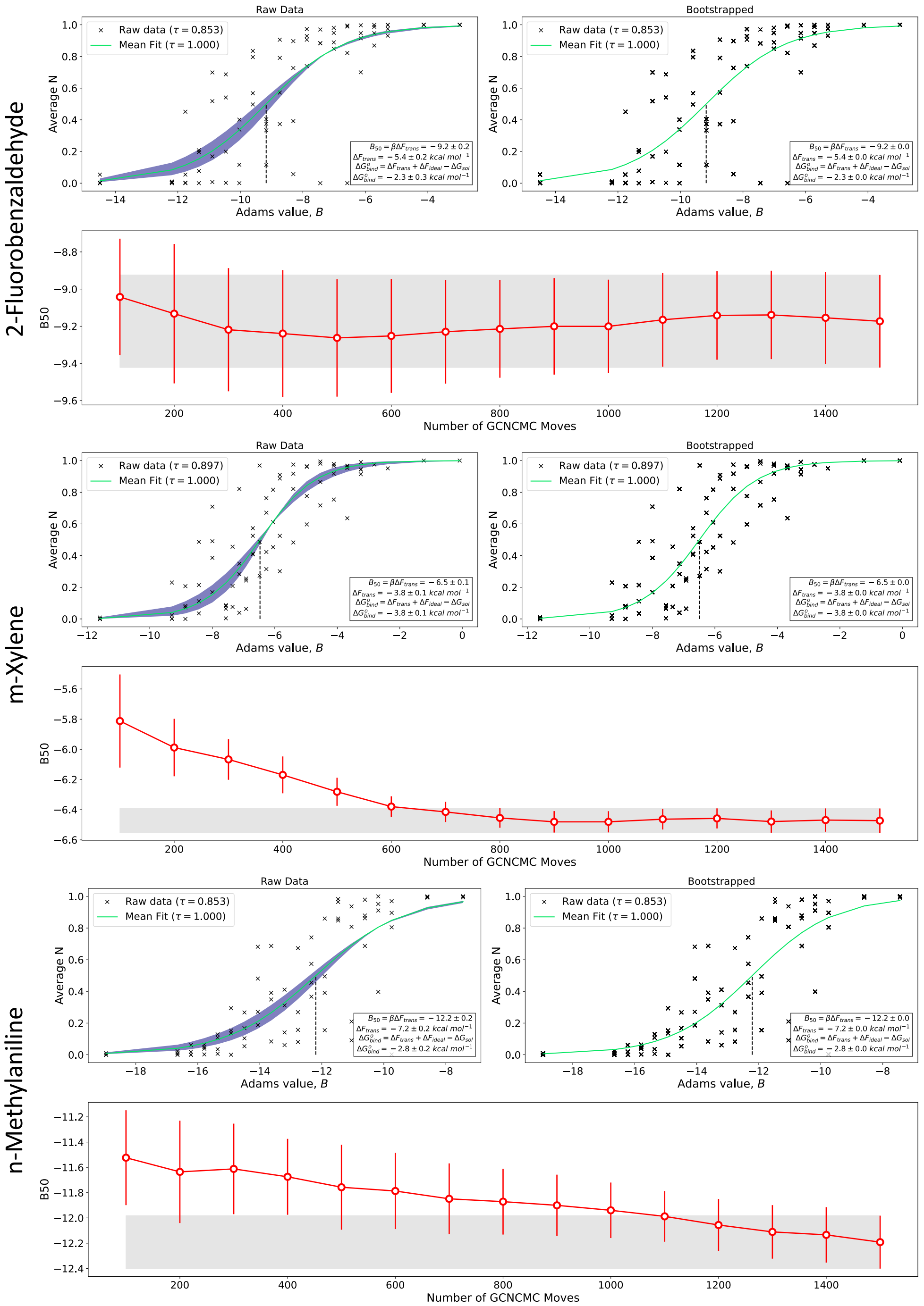

Supplementary Figure 17: Representative titration curves, with and without bootstrapping, and convergence plots for the associated B50 values for examples of good (2-fluorobenzaldehyde) average (m-xylene), and poor (n-methylaniline) performance. The shaded region in the convergence figures corresponds to the standard error of the B50 mean.

2.3.3 ABFE Convergence Studies

Similar to titration calculations, we split our ABFE calculations based on the fraction of MD performed at each lambda and thus plot the calculated free energy as a function of simulation time. Plots for the same ligands as above are shown (Supplementary Figure 18) with the rest provided in the online supplementary data. Here, plots are provided for the complex leg only for direct comparison to titration calculations. Where multiple binding modes need to be considered the associated plot for each binding mode is provided. The average percentage of simulation time required for convergence (where convergence corresponds to bringing the calculated average to within one standard error of the mean) in the complex decoupling legs was 32%.

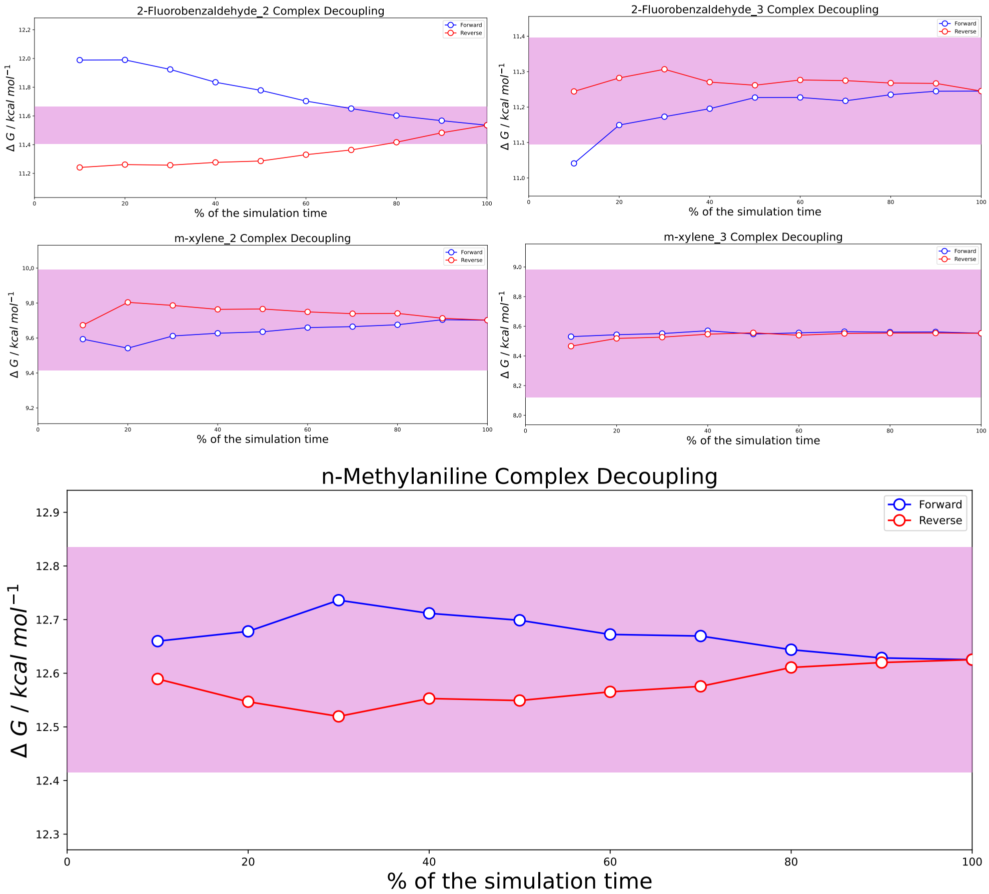

Supplementary Figure 18: Representative convergence plots for the complex decoupling legs for 2-fluorobenzaldehyde, m-xylene, and n-methylaniline. The shaded region in the convergence figures corresponds to the standard error of the mean.

The best, worst, and average cases for the ABFE calculations are also shown in Supplementary Figure 19. Typically, the least well-populated binding modes of ligands are harder to converge.

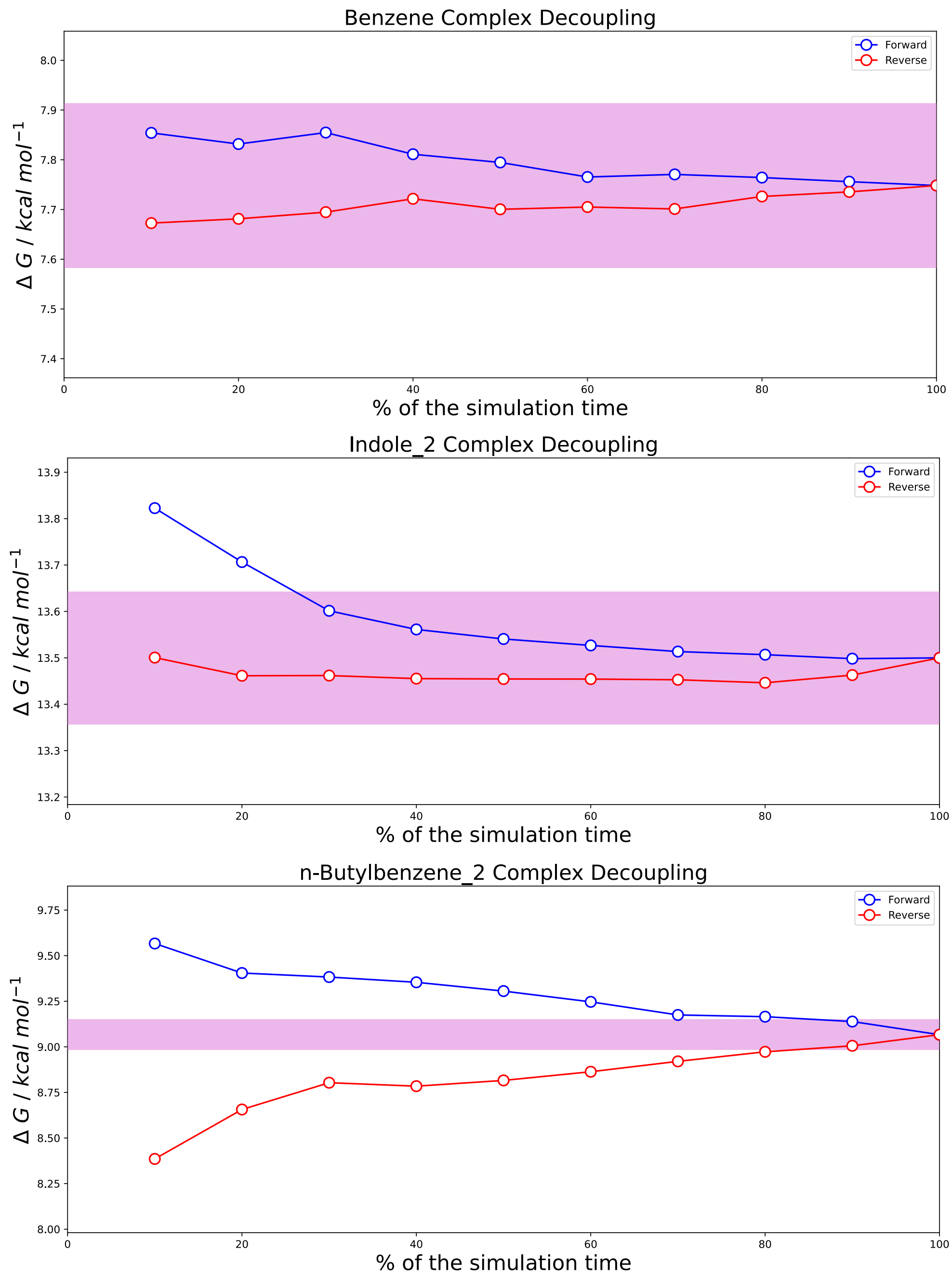

Supplementary Figure 19: Representative convergence plots for the complex decoupling legs for binding modes of Indole and propylbenzene. The shaded region in the convergence figures corresponds to the standard error of the mean.

2.3.4 Comparison of the Two Methods

Finally, to compare the required simulation timescales between GCNCMC and ABFE we have performed the same analysis as above but in terms of the number of force evaluations for T4L99A. This allows for a direct comparison of the amount of computational resources required for each method. It is again worth noting here that ligands that bind in multiple orientations require separate ABFE simulations in both the complex and restraint imposition legs. This is taken into account when comparing the two methods, as for a single ligand binding in two or more orientations (e.g. Toluene) there must be 2 restraint legs and 2 complex legs compared to just the one titration calculation.

For titration calculations, the number of force evaluations per GCNCMC move (including the MD between moves) works out to be 1.14×10^8. This multiplied by 20 B values, as simulated here, is equal to 2.28×10^9 force evaluations. This is the maximum number of force evaluations that could be required. For example, in cases where a deletion move is impossible owing to the binding site being unoccupied then no force evaluations for this particular GCNCMC move are performed. The above calculation assumes the full set of force evaluations are performed for every GCNCMC move and as such constitutes a worst case scenario.

For ABFE restraint legs, the total number of force evaluations per lambda is 2.50×10^6, multiplied by 15 lambdas as simulated here is equal to 3.75×10^7 force evaluations. For complex legs, the total number of force evaluations per lambda is equal to 3.50×10^6, multiplied by 40 lambdas is equal to 1.40×10^8 force evaluations. The solvation leg is not accounted for in these plots as they are a requirement for both methods.

Finally, the total number of force evaluations required for convergence are plotted as a distribution in Supplementary Figure 20 (top) and as cumulative distributions (bottom). Convergence is determined for each ligand as the point where the free energy estimate moves to within one standard error of the mean.

For ABFE calculations we have summed the converged restraint and complex leg force evaluations required for each component of the free energy cycle required to reach convergence. For ligands with multiple binding modes, we further sum the converged force evaluations for each binding mode.

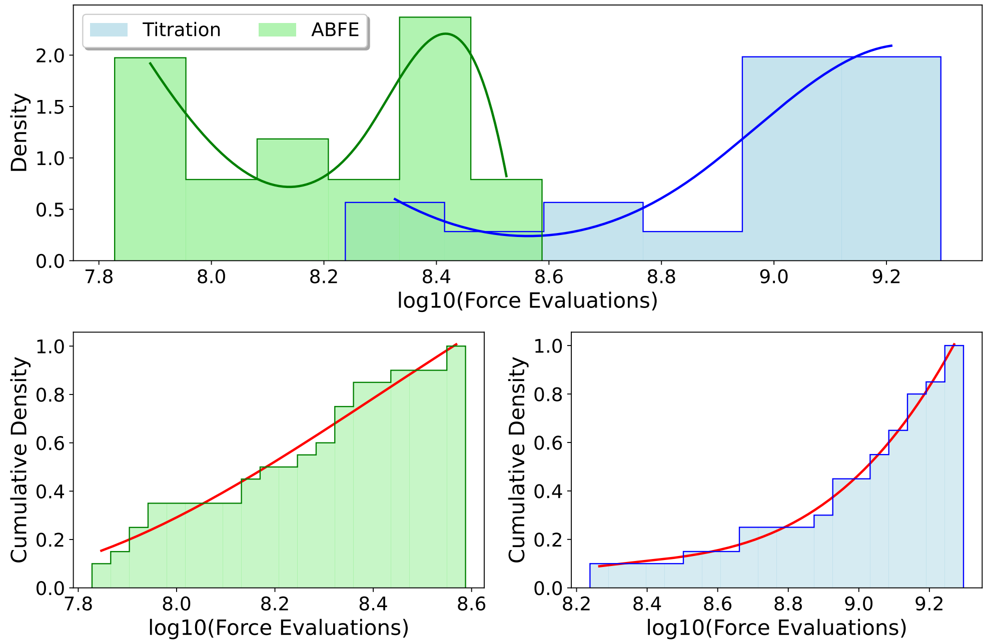

Supplementary Figure 20: **Convergence distributions for T4L99A ABFE and titration calculations.** Top: Distributions of numbers of force evaluations required to achieve converged free energies for both ABFE and GCNCMC titrations. Bottom: Cumulative distributions for both methods. Splines have been fitted to the histograms as a guide to the eye.

It is worth mentioning that both simulation methods and protocols are far from optimized. In the case of titrations, there are many opportunities to improve efficiency which could be further investigated. For example, the effect of switching time (of the GCNCMC moves) on the final free energy estimates should be investigated, as it is possible shorter switching times, and thus fewer force evaluations, are required. Second, the number of B values simulated could also be investigated; indeed the titration calculations of MUP1 used significantly fewer Bs. Lastly, the measurable value, in this case, the average occupancy, converges at different rates depending on the B value. For example, at high B values (corresponding to high concentration) the average N very quickly converges to 1 and vice versa in the low concentration range converging to 0. As such it is likely fewer moves are required at these values of B and a method of detecting convergence on the fly would be valuable here. Similarly, the number of lambda windows and length of simulation used in the ABFE calculations should be investigated with many adaptive ABFE methods now being available[11]. Thus although at first sight, ABFE appears to be more efficient, neither GCNCMC titration nor ABFE protocols have been optimized. Moreover, ABFE methods require *a priori* knowledge of the ligand binding modes, which is not the case for GCNCMC titrations.

2.3.5 Acceptance Rates

While noting that the acceptance rates for GCNCMC moves are a function of B and are likely to be highly system dependent, we wanted to see if there were any discernible trend in the acceptance rates with respect to the size of the ligands. To do this, we find the simulated B value closest to the calculated value of B_{50} and plot the acceptance rate calculated from all the GCNCMC moves at this B value. As B_{50} corresponds to the equilibrium between the reference solution and the binding site, this B value maximizes the number of accepted moves. The calculated acceptance rates for both T4L99A and MUP1 are shown in Supplementary Figure 21. Upon initial inspection, it would appear there is an inverse correlation between acceptance rate and heavy atom count. These results will need further investigation but it seems that, as expected, acceptance probabilities decrease with increasing ligand size and complexity.

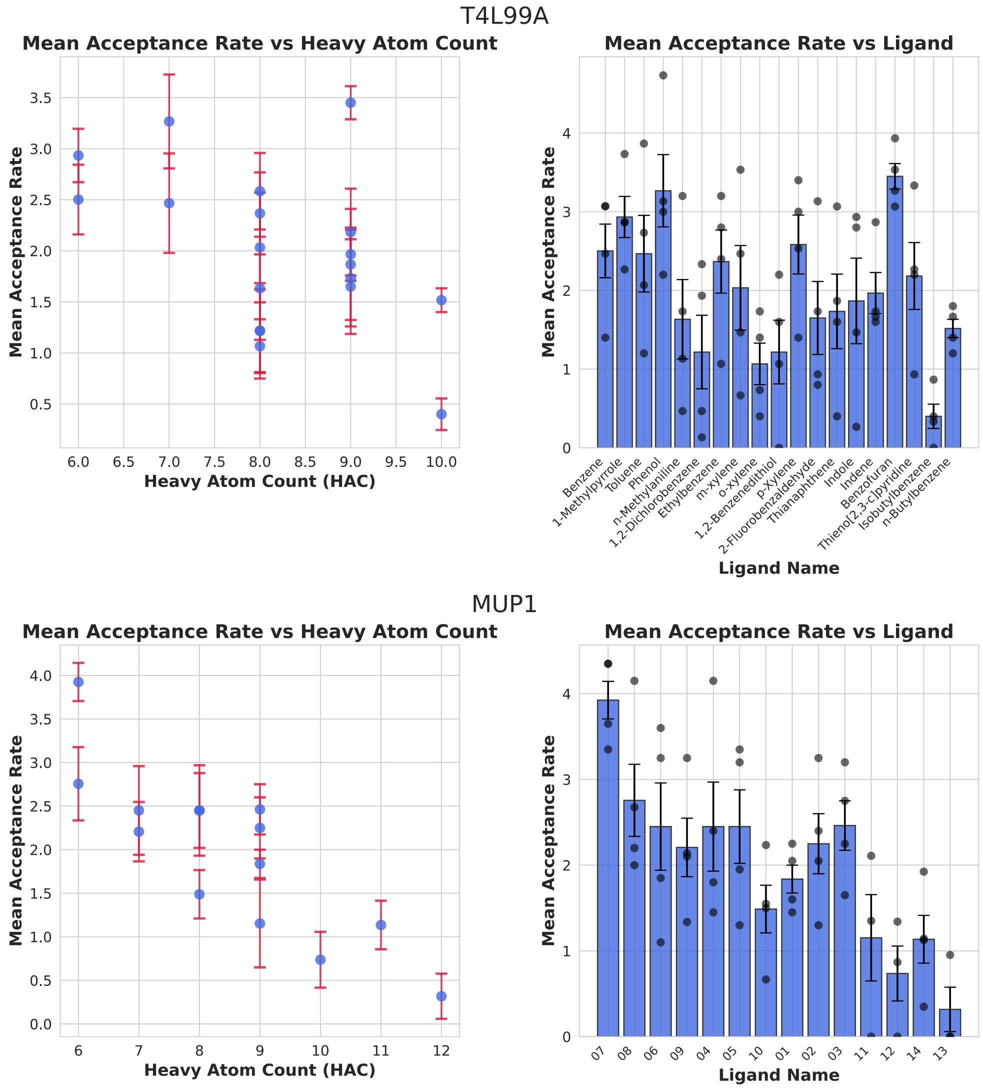

Supplementary Figure 21: **Percentage acceptance rate of GCNCMC moves as a function of ligand heavy atom count.** Top: T4L99A, Bottom: MUP1. Acceptance rates versus ligand name are ordered in terms of increasing heavy atom count.

2.3.6 p-Xylene-Val111 case

As mentioned in the main manuscript, p-xylene binding to T4L99A presents a unique challenge in that upon binding the Val111 sidechain flips from a trans to the gauche(−) conformation (Supplementary Figure 22). If this rotamer flip is not sampled in a free energy calculation, it is likely that the final free energy estimate is erroneous as the

relevant configurational space is not sampled. Indeed, it has been shown previously that binding affinities calculated using an *apo* state will lead to a free energy estimate which is too unfavorable. This is because of the bound ligand and the trans side chain conformation causing unfavorable clashes.[12–14] Conversely, binding of the ligand to the *holo* protein state (g-) can lead to overly favorable free energy estimates as the appropriate Val111 conformation is already in place meaning the reorganization contribution to the free energy is missed. Many enhanced sampling methods have been designed to sample this kind of movement in alchemical free energy calculations but often require some prior knowledge of its existence.[14–16]

In our GCNCMC titration calculations we predict a binding affinity of -4.55 kcal mol^{-1} which is in very good agreement with the experimental value (-4.67 kcal mol^{-1}). This is surprising as it would be expected that the calculated free energy be more unfavorable.

It was hoped that protein side chain transitions would be inherently sampled through constant insertion and deletion moves. In previous studies, we have seen water insertion and deletion move drive configurational change of a bound ligand.[1] However, despite the good agreement with experiment, this was not the case here. To show this, we take all the frames from simulations that returned an average occupancy of 0.4-0.6 and measure the Val111 Chi1 dihedral angle. As indicated by Supplementary Figure 22, almost all the frames sampled the trans conformation observed in the *apo* crystal structure which was used as a starting point for these simulations. This is in line with other alchemical methods which struggle to sample this side chain reorganization.

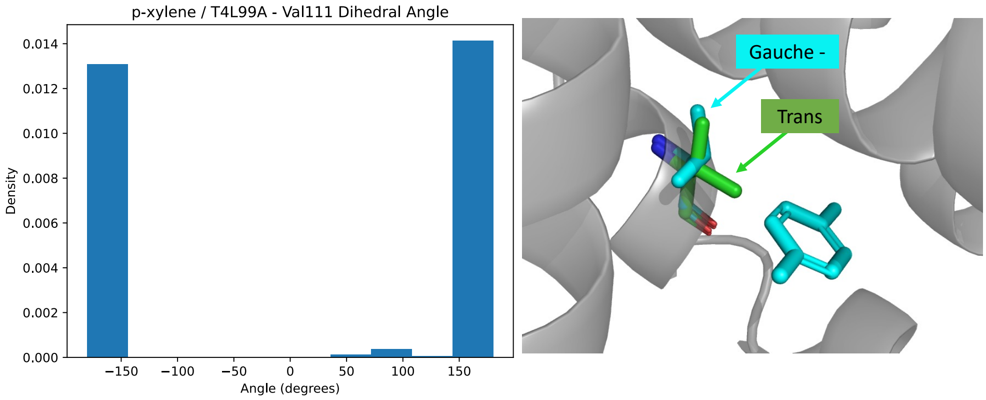

Supplementary Figure 22: Left: Sampled Val111 Chi1 dihedral angles. Right: The two Val111 rotomers; in complex with p-xylene, the Valine flips from trans to gauche-.

One could, in principle, couple the growth of the Val111 side chain to the ligand insertion moves. This does, however, imply prior knowledge of this side chain conformational change, and is therefore not a satisfactory general solution. We are currently investigating the combination of enhanced sampling of protein side chains combined with GCNCMC ligand moves, as a general, system agnostic, solution to this problem.

2.4 MUP1

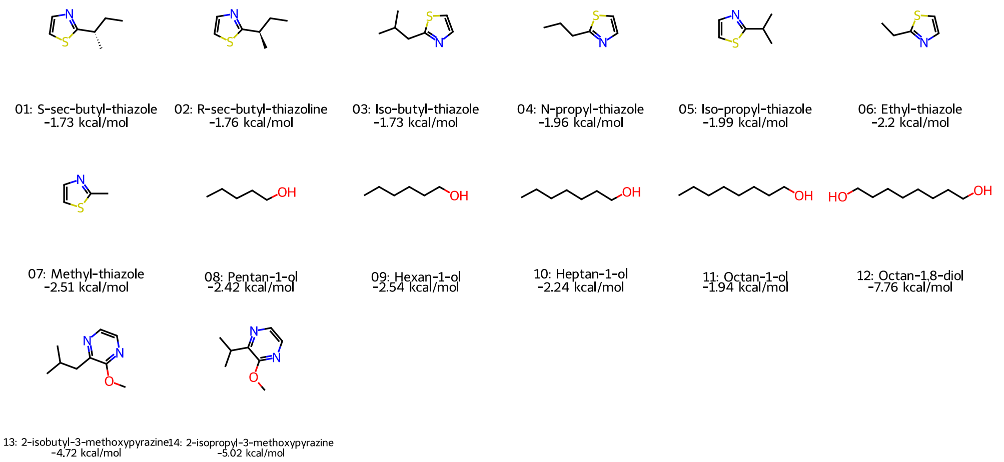

Supplementary Figure 23: MUP1 ligands and their calculated values of excess chemical potential, μ'. Error bars are on the order of 0.05-0.10 kcal mol^{-1} and can be found in the Supplementary Data.

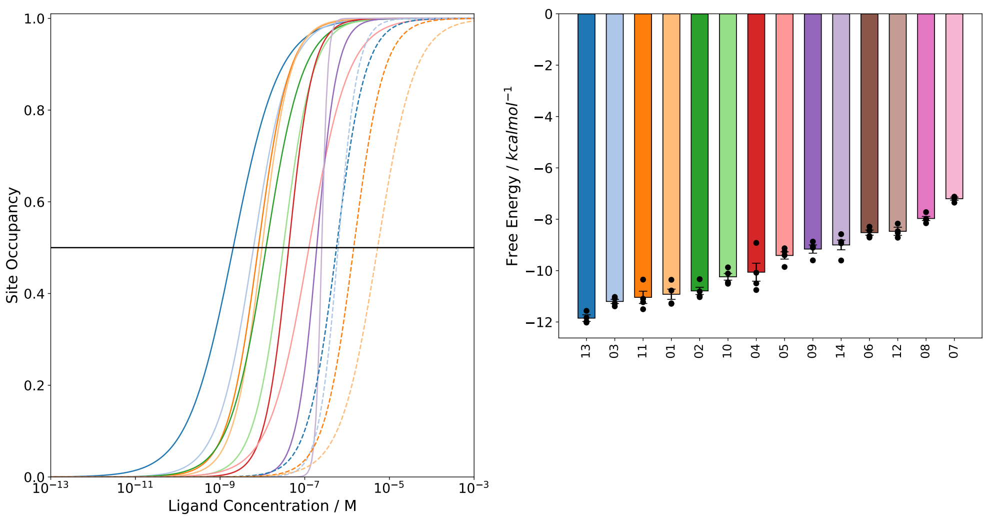

Supplementary Figure 24: **Titration curves for ligands binding to MUP1.** Calculated free energies are derived from the ligand concentration which gives 50% bound occupancy (K_{D}). The error is the standard error of the mean of four repeats.

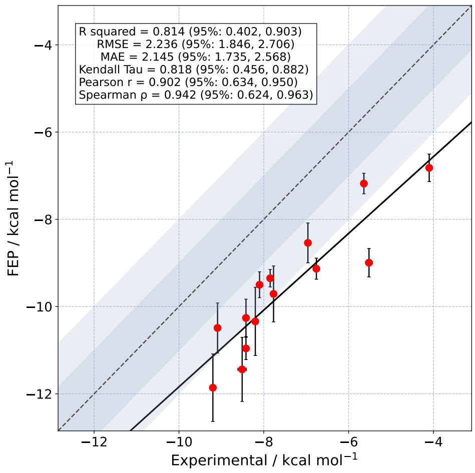

Supplementary Figure 25: **Calculated $\Delta G^{\ominus}$ from FEP calculations vs. experimental data for MUP1.** The error on the ABFE results are the standard error of the mean of 4 individual repeats.

3 Supplementary Methods

3.1 ABFE Calculations

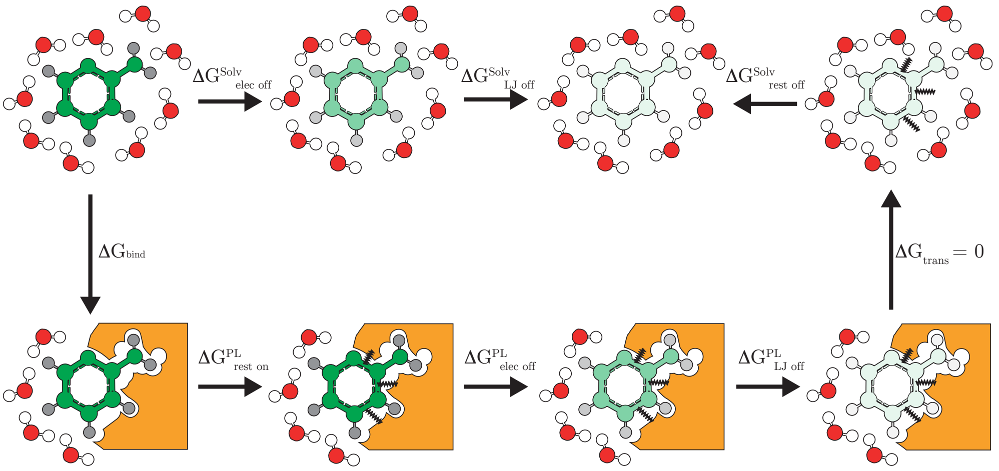

Supplementary Figure 26: ABFE Thermodynamic Cycle

To validate the free energies obtained by titration, we use a more traditional FEP approach.

For each ligand binding to βCD, we selected the most stable pose for each of the two binding modes (primary and secondary) from our GCNCMC simulations as the starting coordinates for the free energy calculations. Two repeats for each binding mode were performed, giving a total of four simulations per ligand. As we found it difficult to define stable Boresch restraints for this host-guest system, we used a spherical flat bottom restraint, with a radius of 5 Å and a force constant of 0.6 kcal mol^{-1} Å^{-2}, to keep the ligands bound. The ligands were then decoupled over 40 lambdas recording potential energy samples every 3 ps. In total, 1000 samples were recorded per lambda giving a total simulation time per lambda of 3 ns. The bound leg free energy of the two binding modes was calculated individually using MBAR and combined using a Boltzmann average. The analytical standard state correction for the restraint was calculated as -0.68 kcal mol^{-1}. The final free energy was calculated according to the thermodynamic cycle in Supplementary Figure 26, reusing the excess chemical potential for the solvent leg.

For T4L99A and MUP1 we selected the most stable poses from our GCNCMC simulations as the starting coordinates for the FEP calculations. For ligands where significant multiple binding modes ($\geq$10% population) were identified in titrations, a full FEP calculation was performed for each mode. This highlights how the number of simulations can quickly grow for fragment molecules that bind in multiple orientations. For each mode, Boresch restraints[17] were used to keep the ligand bound using a force constant of 10 kcal mol^{-1} Å^{-2} for distance restraints and 10 kcal mol^{-1} rad^{-2} for angle and dihedral restraints. The individual restraint atoms were chosen automatically using MDRestraints Generator[18]. To calculate the restraint contribution to the free energy, the restraints were slowly applied to the fully coupled ligand over 15 lambda values. Each lambda recorded 1000 samples at 3 ps intervals for a total of 3 ns per lambda. The analytical standard state correction was calculated using:

$$\Delta G^{\circ}_{\text{restr, off}} = -k_B T \ln \left[\frac{8\pi^2 V^{\circ}}{r_0^2 \sin\theta_{\text{A},0} \sin\theta_{\text{B},0}} \frac{\left(K_{\text{r}} K_{\theta_{\text{A}}} K_{\theta_{\text{B}}} K_{\phi_{\text{A}}} K_{\phi_{\text{B}}} K_{\phi_{\text{C}}}\right)^{1/2}}{(2\pi kT)^3} \right] \tag{62}$$

where r_0, θ_A, and θ_B are the reference values for the distance and two angle restraints. K_X are the force constants for X restraint.

For the bound leg, using the same restraints, the ligands were decoupled over 40 lambdas recording a potential energy sample every 2.5 ps. A total of 2000 samples were collected per lambda giving a simulation time of 5 ns per lambda. The free energy of the bound leg was then calculated using MBAR. The final free energy estimate was calculated using the thermodynamic cycle shown in Supplementary Figure 26. The final free energy estimate for ligands with multiple binding modes was calculated using a Boltzmann average of all the calculated modes. Finally, symmetry corrections were applied where appropriate. Full data can be found in the Supplementary Data.

References

[1] Melling, O. J., Samways, M. L., Ge, Y., Mobley, D. L. & Essex, J. W. Enhanced Grand Canonical Sampling of Occluded Water Sites Using Nonequilibrium Candidate Monte Carlo. *Journal of Chemical Theory and Computation* **19**, 1050–1062 (2023). URL https://doi.org/10.1021/acs.jctc.2c00823.

[2] Nilmeier, J. P., Crooks, G. E., Minh, D. D. L. & Chodera, J. D. Nonequilibrium candidate Monte Carlo is an efficient tool for equilibrium simulation. *Proceedings of the National Academy of Sciences* **108**, E1009–E1018 (2011). URL https://www.pnas.org/content/108/45/E1009.

[3] Fass, J. *et al.* Quantifying Configuration-Sampling Error in Langevin Simulations of Complex Molecular Systems. *Entropy* **20**, 318 (2018). URL https://www.mdpi.com/1099-4300/20/5/318.

[4] Samways, M. L., Bruce Macdonald, H. E. & Essex, J. W. grand: A Python Module for Grand Canonical Water Sampling in OpenMM. *Journal of Chemical Information and Modeling* **60**, 4436–4441 (2020). URL https://doi.org/10.1021/acs.jcim.0c00648. Publisher: American Chemical Society.

[5] Ross, G. A., Bruce Macdonald, H. E., Cave-Ayland, C., Cabedo Martinez, A. I. & Essex, J. W. Replica-Exchange and Standard State Binding Free Energies with Grand Canonical Monte Carlo. *Journal of Chemical Theory and Computation* **13**, 6373–6381 (2017). URL https://doi.org/10.1021/acs.jctc.7b00738.

[6] Ross, G. A., Rustenburg, A. S., Grinaway, P. B., Fass, J. & Chodera, J. D. Biomolecular Simulations under Realistic Macroscopic Salt Conditions. *The Journal of Physical Chemistry B* **122**, 5466–5486 (2018). URL https://doi.org/10.1021/acs.jpcb.7b11734.

[7] Shirts, M. R., Bair, E., Hooker, G. & Pande, V. S. Equilibrium Free Energies from Nonequilibrium Measurements Using Maximum-Likelihood Methods. *Physical Review Letters* **91**, 140601 (2003). URL https://link.aps.org/doi/10.1103/PhysRevLett.91.140601.

[8] Ross, G. A., Bodnarchuk, M. S. & Essex, J. W. Water Sites, Networks, And Free Energies with Grand Canonical Monte Carlo. *Journal of the American Chemical Society* **137**, 14930–14943 (2015). URL https://doi.org/10.1021/jacs.5b07940.

[9] Ross, G. A. *et al.* Enhancing Water Sampling in Free Energy Calculations with Grand Canonical Monte Carlo. *Journal of Chemical Theory and Computation* **16**, 6061–6076 (2020). URL https://doi.org/10.1021/acs.jctc.0c00660.

[10] Träger, S. *et al.* CLoNe: automated clustering based on local density neighborhoods for application to biomolecular structural ensembles. *Bioinformatics* **37**, 921–928 (2021). URL https://doi.org/10.1093/bioinformatics/btaa742.

[11] Clark, F., Robb, G. R., Cole, D. J. & Michel, J. Automated Adaptive Absolute Binding Free Energy Calculations. *Journal of Chemical Theory and Computation* **20**, 7806–7828 (2024). URL https://doi.org/10.1021/acs.jctc.4c00806.

[12] Baumann, H. M., Gapsys, V., de Groot, B. L. & Mobley, D. L. Challenges Encountered Applying Equilibrium and Nonequilibrium Binding Free Energy Calculations. *The Journal of Physical Chemistry B* **125**, 4241–4261 (2021). URL https://doi.org/10.1021/acs.jpcb.0c10263.

[13] Mobley, D. L. *et al.* Predicting absolute ligand binding free energies to a simple model site. *Journal of Molecular Biology* **371**, 1118–1134 (2007).

[14] Mobley, D. L., Chodera, J. D. & Dill, K. A. Confine-and-Release Method: Obtaining Correct Binding Free Energies in the Presence of Protein Conformational Change. *Journal of Chemical Theory and Computation* **3**, 1231–1235 (2007). URL https://doi.org/10.1021/ct700032n.

[15] Suruzhon, M., Bodnarchuk, M. S., Ciancetta, A., Wall, I. D. & Essex, J. W. Enhancing Ligand and Protein Sampling Using Sequential Monte Carlo. *Journal of Chemical Theory and Computation* **18**, 3894–3910 (2022). URL https://doi.org/10.1021/acs.jctc.1c01198.

[16] Burley, K. H., Gill, S. C., Lim, N. M. & Mobley, D. L. Enhancing Sidechain Rotamer Sampling Using Non-Equilibrium Candidate Monte Carlo. *Journal of chemical theory and computation* **15**, 1848–1862 (2019). URL https://www.ncbi.nlm.nih.gov/pmc/articles/PMC6537864/.

[17] Boresch, S., Tettinger, F., Leitgeb, M. & Karplus, M. Absolute Binding Free Energies: A Quantitative Approach for Their Calculation. *The Journal of Physical Chemistry B* **107**, 9535–9551 (2003). URL https://doi.org/10.1021/jp0217839.

[18] Alibay, I., Magarkar, A., Seeliger, D. & Biggin, P. C. Evaluating the use of absolute binding free energy in the fragment optimisation process. *Communications Chemistry* **5**, 1–13 (2022). URL https://www.nature.com/articles/s42004-022-00721-4.
